# Alpha-Adrenergic Mechanisms in the Cardiovascular Hyperreactivity to Norepinephrine-Infusion in Essential Hypertension

**DOI:** 10.3389/fendo.2022.824616

**Published:** 2022-07-22

**Authors:** Lisa-Marie Walther, Roland von Känel, Nadja Heimgartner, Claudia Zuccarella-Hackl, Guido Stirnimann, Petra H. Wirtz

**Affiliations:** ^1^ Biological Work and Health Psychology, University of Konstanz, Konstanz, Germany; ^2^ Centre for the Advanced Study of Collective Behaviour, University of Konstanz, Konstanz, Germany; ^3^ Department of Consultation-Liaison Psychiatry and Psychosomatic Medicine, University Hospital Zurich, University of Zurich, Zurich, Switzerland; ^4^ Division of Clinical Psychology and Psychotherapy, University of Basel, Basel, Switzerland; ^5^ Department of Visceral Surgery and Medicine, University Hospital Inselspital and University of Bern, Bern, Switzerland

**Keywords:** essential hypertension, norepinephrine-infusion, alpha-adrenergic receptor blockade, phentolamine, cardiovascular reactivity

## Abstract

**Aims:**

Essential hypertension (EHT) is characterized by cardiovascular hyperreactivity to stress but underlying mechanism are not fully understood. Here, we investigated the role of α-adrenergic receptors (α-AR) in the cardiovascular reactivity to a norepinephrine (NE)-stress reactivity-mimicking NE-infusion in essential hypertensive individuals (HT) as compared to normotensive individuals (NT).

**Methods:**

24 male HT and 24 male NT participated in three experimental trials on three separate days with a 1-min infusion followed by a 15-min infusion. Trials varied in infusion-substances: placebo saline (Sal)-infusions (trial-1:Sal+Sal), NE-infusion without (trial-2:Sal+NE) or with non-selective α-AR blockade by phentolamine (PHE) (trial-3:PHE+NE). NE-infusion dosage (5*µ*g/ml/min) and duration were chosen to mimic duration and physiological effects of NE-release in reaction to established stress induction protocols. We repeatedly measured systolic (SBP) and diastolic blood pressure (DBP) as well as heart rate before, during, and after infusions.

**Results:**

SBP and DBP reactivity to the three infusion-trials differed between HT and NT (*p*’s≤.014). HT exhibited greater BP reactivity to NE-infusion alone compared to NT (trial-2-vs-trial-1: *p*’s≤.033). Group differences in DBP reactivity to NE disappeared with prior PHE blockade (trial-3: *p*=.26), while SBP reactivity differences remained (trial-3: *p*=.016). Heart rate reactivity to infusion-trials did not differ between HT and NT (*p*=.73).

**Conclusion:**

Our findings suggest a mediating role of α-AR in DBP hyperreactivity to NE-infusion in EHT. However, in SBP hyperreactivity to NE-infusion in EHT, the functioning of α-AR seems impaired suggesting that the SBP hyperreactivity in hypertension is not mediated by α-AR.

## 1 Introduction

Arterial hypertension is a major risk factor for cardiovascular disease (CVD) (1). About 95% of individuals with arterial hypertension are diagnosed with “essential” hypertension (EHT), meaning that their elevated blood pressure (BP) is not secondary to a medical cause (2). Hyperactivity of the sympathetic-nervous-system may play a mediating role in CVD risk with hypertension (3). In particular, greater cardiovascular reactivity to acute mental stress, or hyperreactivity, respectively, as seen in hypertensive and hypertension-prone individuals (4, 5) has been associated with future cardiovascular risk (6). However, the mechanisms underlying greater cardiovascular reactivity to acute mental stress in EHT are not fully understood.

Cardiovascular reactivity to acute mental stress is primarily mediated by the catecholamines epinephrine (EPI) and norepinephrine (NE) during activation of the sympathetic-adrenal-medullary axis (7). With respect to *circulating catecholamine concentrations*, hypertensive individuals (HT) show elevated plasma catecholamine levels under resting conditions (8). However, most studies investigating the catecholamine reactivity to acute mental stress found no differences between HT and normotensive individuals (NT) in both, NE and EPI reactivity (9). This indicates that HT show a normal, i.e. normotensive, catecholamine stress reactivity, but on a consistently higher level due to elevated basal catecholamine levels. To investigate potential differences in *effects of catecholamines*, studies compared cardiovascular reactivity to catecholamine-infusion between HT and NT. With respect to NE-infusion, EHT showed higher cardiovascular reactivity, lower threshold doses (i.e. infusion dose necessary to initiate BP increases), and/or lower pressor doses (i.e. infusion dose necessary to increase mean BP about 20 mmHg) (10, 11). In contrast, cardiovascular reactivity to EPI-infusion in HT as compared to NT is less conclusive (12, 13). Notably, infusion of EPI is confounded by concomitant NE-release as EPI-infusion dose-dependently induces plasma increases of both, EPI and NE, whereas NE-infusion however increases NE but not EPI (14). Taken together, catecholamine release in reaction to stress does not seem to differ between HT and NT but infusion studies suggest higher cardiovascular reactivity to NE-infusion in hypertension.

Hitherto, the mechanisms underlying the higher cardiovascular reactivity to NE-infusion in individuals with EHT are largely unknown. Catecholamine effects on the cardiovascular system are mediated *via* adrenergic receptors (AR) (15). While β-AR do not seem to play a major role in cardiovascular reactivity differences to NE-infusion (16) or mental stress (17) between HT and NT, the functioning of α-AR has hardly been studied in the context of hypertension so far. *Under steady-state conditions* findings are mixed. While some studies detected increased α_1_-mediated vasoconstriction in EHT to selective α-adrenergic agonists or antagonists suggesting increased α-AR sensitivity (18, 19), other studies could not find alterations in α-AR functioning in HT (20, 21). *In reaction to NE-infusion*, SBP and DBP reactivity in NT were blunted after selective α_1_-, selective α_2_-, and non-selective α-AR blockade (22, 23), with strongest effects after α_1_-AR blockade (22) while there were no effects of α-AR blockade on heart rate (HR) reactivity (22, 23). In HT, cardiovascular reactivity to NE-infusion after α-AR blockade has been only investigated using selective α_1_-AR blockade. Here, two studies in HT only found diminished mean arterial BP (MAP) reactivity (24) as well as diminished DBP responsiveness (25) to NE-infusion after selective α_1_-AR blockade as compared to placebo, while HR reactivity to NE-infusion did not differ between α_1_-AR blockade and placebo condition (24). Taken together, in the context of cardiovascular reactivity to either stress or catecholamine-infusion, studies using non-selective α-AR blockade are lacking in HT and α-AR functioning has not yet been compared between HT and NT.

To shed light on the role of α-AR, we investigated for the first time cardiovascular reactivity to a NE-stress reactivity-mimicking standardized NE-infusion with and without prior non-selective α-AR blockade by phentolamine (PHE) in EHT as compared to NT. Using a within-subject design, we repeatedly measured BP and HR before, during, and after infusion of NE with and without prior PHE-infusion or saline (Sal). We expected BP hyperreactivity to NE-infusion in HT as compared to NT but no differences in HR reactivity. Moreover, we hypothesized BP hyperreactivity to NE-infusion in HT to be modulated by α-AR mechanisms. We controlled for potential confounders of reactivity and recovery kinetics.

## 2 Materials and Methods

### 2.1 Study Participants

This study is part of a larger project investigating the effects of α-AR blockade on NE-induced physiological stress reactivity (23, 26, 27) conducted between 2007 and 2012. Here, we report for the first time results of the clinical part of the project where we extended recruitment to HT in addition to NT. The infusion procedure was identical with the basic research part of the project (23, 26, 27). The study was formally approved by the Ethics Committee of the State of Bern, Switzerland, and the Swiss Agency for Therapeutic Products (Swissmedic), and conducted in accordance with the Declaration of Helsinki principles. All participants provided written informed consent prior to the study and were financially compensated for their participation with 120 CHF for each of three study days.

With the aid of the Swiss Red Cross of the Canton of Bern and the Clinical Investigation Unit (CIU) of the University Hospital of Bern/Inselspital Bern, we recruited hypertensive and normotensive otherwise healthy and medication-free men between 30 and 66 years. Whenever possible, for each recruited HT we recruited a NT of similar age on a case-by-case basis. In detail, members of our study team accompanied the mobile blood-donation unit of the Swiss Red Cross that routinely assesses BP prior to blood donation. Male blood donors with elevated or normal BP expressing interest in study participation were asked to provide an initial home BP diagnostic as part of the assessment of EHT (see below) and were invited for a clinical screening to verify study eligibility. Participation was restricted to male subjects, in particular because of gender differences in cardiovascular responsiveness to stimulation by infusion of NE (28) and of the α-AR agonist phenylephrine, with higher reactivity in men as compared to women (29). Moreover, reactivity to inhibition differed between men and women with men showing higher responsiveness to α-AR blockade by PHE (30).

Specific exclusion criteria as verified by a structural interview as part of the clinical screening included: any regular or current prescribed or non-prescribed medication intake, psychopathology or psychiatric diseases, respectively, alcohol abuse, smoking and/or illicit drug use, any heart diseases, varicosis and thrombotic diseases, elevated blood sugar levels and diabetes, elevated cholesterol levels, liver and renal diseases, chronic obstructive pulmonary disease, allergies and atopic diathesis, rheumatic diseases, human immunodeficiency virus, cancer, chronic pain, sleep disturbances, thyroid diseases and current infectious diseases. Furthermore, participants provided blood samples for the routine assessment of serum creatinine, sodium, potassium, HbA1c and total cholesterol/high-density lipoprotein ratio to identify potential cases with secondary hypertension; no participant was diagnosed with secondary hypertension and all participants were included (**Supplementary Table 1**).

### 2.2 Assessment of Essential Hypertension

For the assessment of EHT and normotension, we applied a two-step assessment procedure that, in combination with the blood examination, aimed at excluding secondary hypertension as well as white coat and masked hypertension in all eligible participants. For both, home BP and screening BP assessment, we followed the respective recommendations for blood pressure measurements of the European Society of Hypertension (ESH) (31). (1) *Home BP assessment*. Following written instructions, each candidate interested in study participation provided an initial BP diagnostic by home assessment using sphygmomanometry (Omron M6; Omron Healthcare Europe B.V., Hoofddorp, Netherlands) (32, 33). Home BP measurements were to be obtained in a seated position after a minimum of 15 min rest twice per day (once in the morning and once in the evening) on up to 3 separate days. Based on the up to six home BP measurements, we computed the average home BP. Participants were preliminarily categorized as hypertensive following the European Society of Hypertension recommendations for home BP measurements (hypertension: average home assessed SBP≥135 mmHg and/or average home assessed DBP≥85 mmHg) (34). Correspondingly, participants were preliminarily considered normotensive if their average home assessed SBP was below 135 mmHg and their average home assessed DBP below 85 mmHg. (2) *Screening BP assessment.* Preliminary home BP classification was extended by the mean of two additional seated BP measurements during the clinical screening. For these measurements, BP assessment was performed by trained personnel using automated sphygmomanometry (Eagle 4000, Software Version 6F, Marquette Hellige GmbH, Freiburg) after resting periods of at least 5 min. For categorization into HT and NT according to their screening BP, we applied the World Health Organization/International Society of Hypertension definition and considered participants as HT with average screening SBP≥140 mmHg and/or average screening DBP≥90 mmHg (35). Participants were considered as NT if their average screening SBP and DBP was below 140 mmHg and 90 mmHg, respectively (35). Notably, only participants with consistent classification as HT or NT in both, home and screening BP assessment, were eligible for study participation. Moreover, due to ethical considerations, individuals with hypertension grade 3 (34) were not allowed to participate in this study.

Based on our *a priori* sample size calculation (see below) and the planned Latin Square Design (see below), we aimed at recruiting 24 HT and 24 NT who met all inclusion criteria.

**Figure 1:**
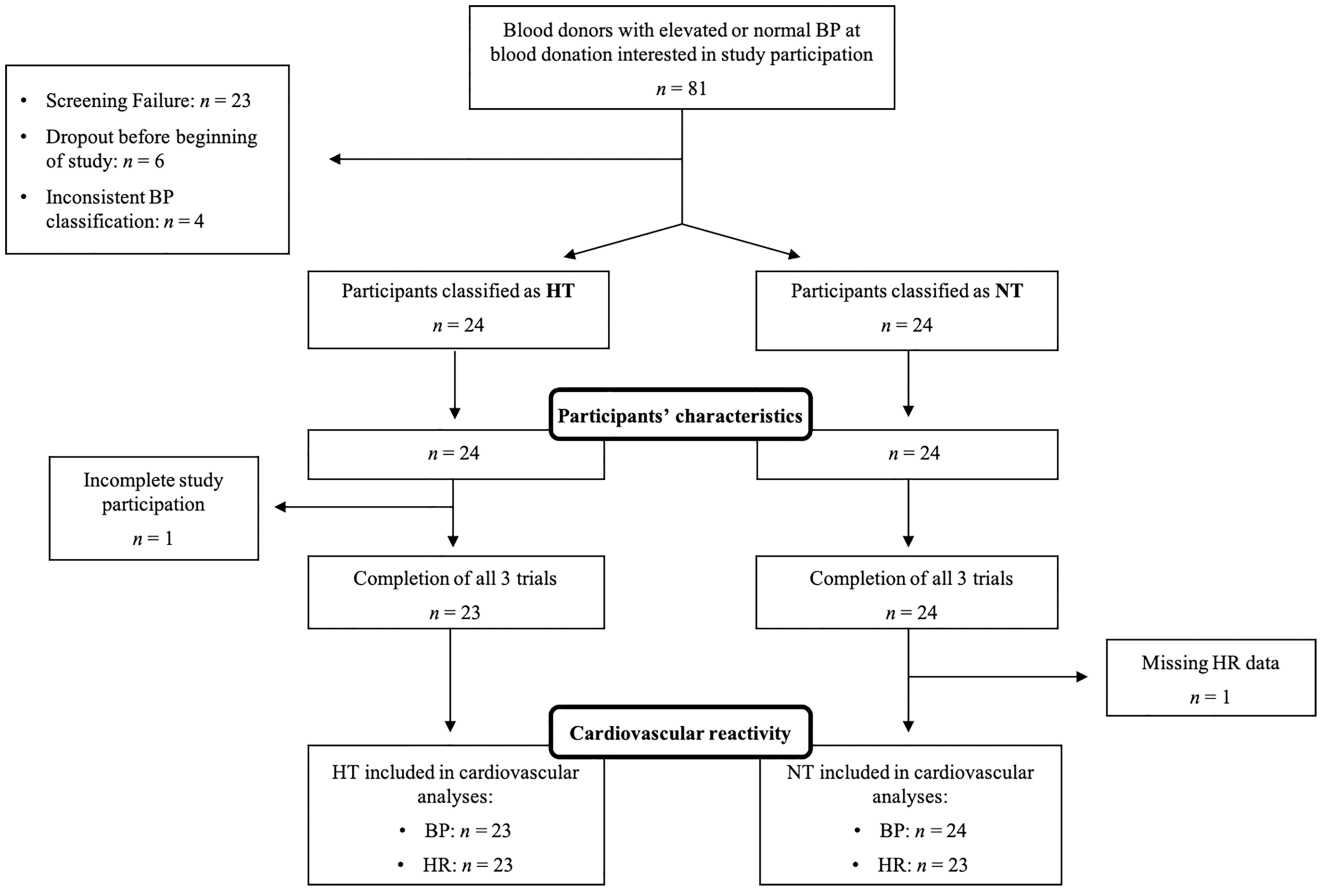
Participant flow chart. BP, blood pressure; HT, hypertensive participants; HR, heart rate; *n*, sample size; NT, normotensive participants.

Since one hypertensive participant completed only one of the three trials (trial 3), a total of 47 participants (23 HT and 24 NT) completed all three trials (see **Figure 1** for participants’ flow through the study). Four HT and six NT did not provide home BP measurements. To maintain a two-step BP assessment procedure, we therefore substituted these missing home BP measurements by the mean of the resting study BP readings of the second and third study day (see below).

### 2.3 Study Design and Procedure

**Figure 2:**
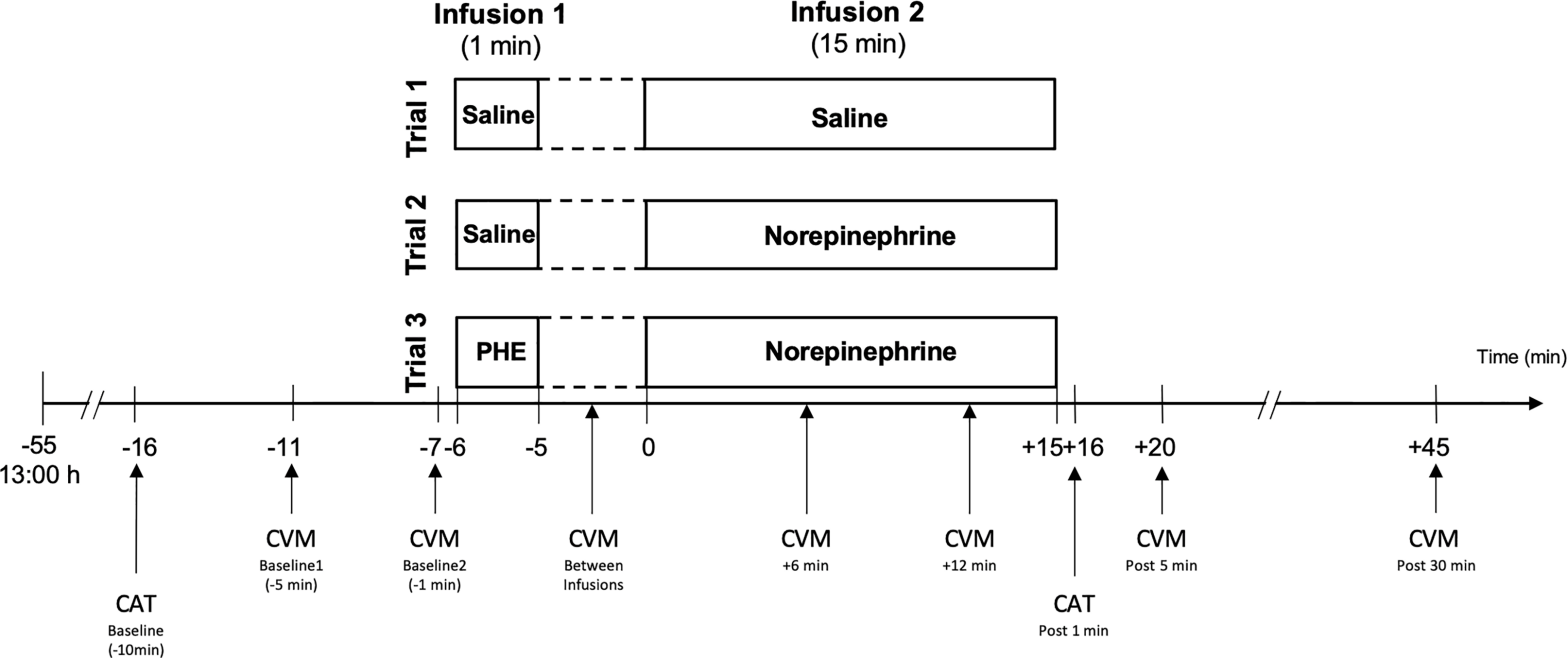
Infusion procedure and measurements. Infusion dosages: Norepinephrine: 75 *µ*g (5 *µ*g/ml/min); PHE: 2.5 mg. CVM, cardiovascular measurement; i.e. blood pressure and heart rate assessment; CAT, catecholamine assessment; PHE, phentolamine; post, post infusion 2.

The study was performed at the CIU of the Inselspital, Bern University Hospital. In a single-blind placebo-controlled within-subject design, all participants completed three experimental trials on three separate days with two sequential standardized infusions, a first 1-min infusion (infusion 1) followed by a second 15-min infusion (infusion 2). We decided for this within-subject design to take into account potential individual differences in infusion reactivity in the different trials. Experimental trials varied in the combination of infused substances as previously described (23). Trial-1 with Sal-only infusion (Sal+Sal) was performed to control for potential effects of the infusion procedure per se and therefore was considered as placebo-condition. Trial-2 (Sal+NE) aimed to test the effects of a NE-stress reactivity-mimicking NE-infusion (see below). Trial-3 (PHE+NE) was designed to test whether potential NE-infusion effects are modulated by α_1_- and α_2_-AR, i.e. non-selective α-AR blockade by PHE (see below). The infusion procedure is depicted in **Figure 2**. Trials were conducted on separate days. In order to control for potential sequence effects of substance infusions, we used a Latin Square Design with the following sequences: 1, 2, 3 (i.e. Sal+Sal on infusion-day 1, Sal+NE on infusion-day 2, PHE+NE on infusion-day 3); 2, 3, 1 (i.e. Sal+NE on infusion-day 1, PHE+NE on infusion-day 2, Sal+Sal on infusion-day 3); 3, 1, 2 (i.e. PHE+NE on infusion-day 1, Sal+Sal on infusion-day 2, Sal+NE on infusion-day 3). The random allocation of study participants was realised by computer software. In order to allow for a sufficient wash-out period for PHE, inter-trial intervals after PHE+NE were one to two weeks. As ethical and safety considerations regarding potential (cardiovascular) side effects of study substances prohibited a double-blind design, only participants but not experimenters were blind to trial substances to rule out that participants’ knowledge and/or beliefs about treatments and treatment effects may affect outcomes *via* the placebo–nocebo phenomenon or premature anticipatory effects (36). A board-certified internist performed all infusions and participants were consistently kept under surveillance in order to be able to immediately handle unexpected medical emergencies or side effects of NE- and/or PHE-infusions (see **Supplemental Material** “Safety aspects of infusion procedure”).

Participants abstained from physical exercise for 24h and from alcohol and caffeinated beverages from the evening before every study day. Moreover, participants were asked to keep a regular sleep-wake rhythm the three nights before each trial, with sleep starting between 22:30h and 24:00h and awakening between 07:00h and 09:00h. Participants arrived at the CIU laboratory at 11:45h where they received a standardized meal. Experimental procedures commenced at 13:00h with a 10-min introduction phase during which the testing procedure was explained. Catheter insertion into the brachial vein of the dominant arm for substance-infusion and into the brachial vein of non-dominant arm for blood sampling followed. After a subsequent 45-min acclimatisation phase, infusion procedures started. The infusion phase started with a 1-min infusion of Sal or PHE (first infusion), followed by a 5-min waiting period. Next, Sal or NE was infused for 15 min (second infusion). The post-infusion phase began after the end of the second infusion. Notably, participants were in supine position lying on a bed for the entire experimental procedure.

### 2.4 Substance Infusion

For NE-infusion in order to mimic effects of NE-stress reactivity NE (Sintetica, SA, Mendrisio, Switzerland) was diluted in Sal and the resulting solution of 5μg/ml was infused with a constant speed of 1 ml/min over a 15-min period (rendering a total of 75 μg NE This dosage was chosen because of earlier studies showing that a dose of 5μg/ml/min NE, yielding plasma levels in excess of 1800 pg/ml, is required to produce measurable cardiovascular effects as elicited in reaction to acute (mental) stress (37). The 15-min infusion interval was chosen based on the duration of the well-established and potent stress induction protocol ‘Trier Social Stress Test’ (TSST) (38) so that our NE-infusion mimics duration and effectiveness (in terms of effects on BP) of NE-release in reaction to acute psychosocial stress by means of the TSST. For α-AR blockade, the non-selective α-adrenergic antagonist (i.e. α_1_- and α_2_-AR blocker) PHE (Regitin®, Novartis Pharma AG, Basel, Switzerland) was diluted in Sal and 5 ml of the resulting solution with 0.5 mg/ml PHE were infused within 1 min (rendering a total of 2.5 mg PHE) according to a pharmacologist’s instruction based on the manufacturer’s recommendation. For the corresponding Sal-infusions in trial-1 and trial-2, we chose identical time intervals of 1 min and 15 min, respectively. We previously demonstrated effectiveness of the infusion dosages and durations used in our study protocol (23, 26, 27). Notably, we favoured infusion of a standardized NE dose over weight-adjusted infusion due to the following: a standardized infusion usually depends on body weight with higher body weight relating to lower infusion reactivity. To control for weight effects, most studies apply weight-adjustment of the infusion dose, in particular if the main variable of interest is unrelated to weight. However, given that HT usually show a higher BMI (2), a weight-adjustment could confound potential group differences in favour of the group with higher BMI. In contrast, infusion of a standardized NE dose rules out this potential confounding and represents a more rigorous approach in groups differing in BMI as a potential higher reactivity in the group with higher BMI cannot result from a higher infusion-dose.

### 2.5 Physiological Measurements

#### 2.5.1 Catecholamines

For EPI and NE assessment, venous blood was drawn into EDTA (ethylenediaminetetraacetic acid)-coated monovettes (Sarstedt, Numbrecht, Germany). Blood samples were taken at baseline, i.e. before the first infusion, and 1 min after the end of the second infusion (see **Figure 2**).

After immediate centrifugation at 2000g and 4°C for 10 min, plasma was stored at - 80°C until analyses. Plasma EPI and NE levels were determined by means of high-pressure liquid chromatography and electrochemical detection after liquid-liquid extraction in the Laboratory for Stress Monitoring, Göttingen, Germany with inter- and intra-assay CVs <5% and a lower detection limit of 12 pg/ml. Undetectable EPI levels were replaced by half the detection limit.

In three HT and one NT, NE and/or EPI levels were missing because of technical problems with high-pressure liquid chromatography.

#### 2.5.2 Cardiovascular Measures

BP and HR were assessed in supine position by trained personnel using automated sphygmomanometry (Eagle 4000, Software Version 6F, Marquette Hellige GmbH, Freiburg). We investigated differences in infusion reactivity between trials based on six timepoints: a baseline measurement, comprising the mean of measurements obtained five and one min before start of the first infusion, one measurement after the first infusion but before the beginning of the second infusion, two measurements during the second infusion (i.e. 6 min and 12 min after the beginning of infusion 2) as well as two post-infusion measurements 5 min and 30 min after the end of the second infusion (see **Figure 2**). Measurement time points were chosen based on previous stress studies showing that cardiovascular measures peak throughout stress exposure and return to pre-stress levels within 10 to 20 mins (39–42).

We had missings in cardiovascular measures due to technical problems: baseline measurement comprised only one instead of the mean of two measurements in one of the three trials for seven HT (trial-1: two HT, trial-2: two HT, trial-3: one HT); baseline HR measurement comprised only one instead of the mean of two measurements in one of the three trials for five participants (trial-1: two NT, trial-2: one HT, trial-3: two HT); in one NT, HR data for trial-3 were missing. One HT did dropout and completed only trial-3.

### 2.6 Statistical Analyses

Data were analysed using SPSS (Version 26) statistical software package for Macintosh (IBM SPSS Statistics, Chicago, Il, USA) and presented as mean±standard error of the mean (SEM). Analyses were two-tailed with the level of significance set at *p*<.05 and *p*-values <.10 interpreted as borderline significant. All data were tested for normal distribution and homogeneity of variance using Kolmogorov-Smirnov and Levene tests. We applied Huynh-Feld-correction where appropriate. Mean resting study BP was calculated from a total of four baseline BP measurements obtained on study days two and three. We calculated MAP based on mean resting study BP readings and the mean of the two screening BP measurements by the formula MAP=2/3*mean DBP+1/3*mean SBP. Body mass index (BMI) was calculated by the formula BMI=kg/m^2^.

To test for *group differences (HT* vs. *NT) in demographic and physiological measures*, we calculated one-factorial analyses of variance (ANOVA) with group (HT vs. NT) as the independent variable and demographic and physiological measures as dependent variables.

To verify *effective PHE application* and to test for *group differences in PHE application*, we tested its known effects on BP and HR. We considered changes in reaction to infusion 1 (trial-1 and trial-2: Sal, trial-3: PHE) by first calculating changes in response to infusion 1 for each trial as difference in SBP, DBP, and HR between levels after the first infusion and baseline levels. Subsequently, we calculated repeated measures ANCOVAs with first-infusion-induced changes in SBP, DBP, or HR of all three trials as repeated dependent variable and group (HT vs. NT) as independent variable while controlling for trial order. We tested for main effects of trial to detect trial differences over all participants and we tested for interactions trial-by-group to detect reactivity differences between HT and NT. *Post-hoc* testing comprised dependent t-tests within each trial separately with pre- and post-infusion 1 measurements.

To test for *trial and group (HT* vs. *NT) differences in baseline catecholamines*, we calculated repeated measures ANCOVAs with baseline catecholamine levels of all trials as repeated dependent variable and group (HT vs. NT) as independent variable controlling for trial order. We tested for main effects of trial to detect trial differences and for main effects of group to detect group differences.

To test for *infusion-induced changes in NE and EPI between trials* and for *differences in infusion-induced changes in NE and EPI between the two groups*, we first calculated infusion-induced changes for each trial as difference in plasma levels between 1 min post second infusion and baseline. Subsequently, we calculated repeated measures ANCOVAs with infusion-induced changes of all three trials as repeated dependent variable and group (HT vs. NT) as independent variable controlling for trial order. We tested for main effects of trial to detect trial differences and for main effects of group as well as interactions trial-by-group to detect group differences. *Post-hoc* tests comprised dependent t-tests between trials (i.e. Sal+Sal vs. Sal+NE, Sal+Sal vs. PHE+NE, Sal+NE vs. PHE+NE). Further, we calculated dependent t-tests for infusion induced changes within each trial separately.

Our *main analyses* comprised testing for *differences in trial infusion reactivity* of BP and HR between HT and NT. We first calculated general linear models (GLM) with the two repeated dependent factors trial (3 trials) and time (6 measurement time-points for SBP, DBP, and HR), and group (HT vs. NT) as independent variable. As *post-hoc* tests, we compared trials pairwise (i.e. Sal+Sal vs. Sal+NE; Sal+Sal vs. PHE+NE; Sal+NE vs. PHE+NE) by repeating the above described GLMs with two trials. Further *post-hoc* analyses comprised separate analyses of each trial alone by means of repeated measures ANCOVAs with repeated SBP, DBP, or HR levels as repeated dependent variable and group (HT vs. NT) as independent variable. Trial order was controlled in all main analyses. Additionally, we controlled for age and BMI to account for potential age- and weight-related differences in NE-infusion reactivity (43, 44) and to rule out a mediating effect of BMI as HT usually show a higher BMI as compared to NT (2).

Complementary main analyses comprised testing for linear associations between cardiovascular trial infusion reactivity and MAP as a continuous measure of hypertension assessment by calculating the same GLMs and repeated measures ANCOVAs with MAP as continuous independent variable instead of group.

We a-priori calculated a sample size of *N*=48 to detect interactions between groups (HT vs. NT) and repeated cardiovascular parameters (6 measurement timepoints) in repeated measures ANOVAs with small to medium effects (*f*=.15) given α=.05, a power of .90. Effect size parameters (*f*) were calculated from partial eta square (*η^2^_p_*) using G*Power for Macintosh (Version 3.1.9.6) and are reported where appropriate (effect size conventions: *f* .10=small, .25=medium, and .40=large).

## 3 Results

### 3.1 Participants’ Characteristics

**Table 1** depicts the characteristics of our final study sample comprising 24 HT and 24 NT. As expected, HT had significantly higher average SBP, DBP, and MAP compared to NT (*p*’s<.001) and HT also had higher BMI (*p*=.013). As intended, the two groups did not differ in terms of age as well as resting HR (*p*’s≥.44).

**Table 1:** Participants’ characteristics.

	Normotensives (*n* = 24)	Hypertensives (*n* = 24)	*p*
Age (years)	54.17 ± 1.88 (33 – 66)	54.46± 1.29 (38 – 64)	.90
BMI (kg/m^2^)	24.07 ± 0.43 (20.73 – 29.04)	25.78 ± 0.50 (21.29 – 31.93)	**.013**
Home BP	*n* = 18 [*n* = 24]	*n* = 20 [*n* = 24]	
SBP (mmHg)	122.23 ± 1.89 [119.68 ± 1.78] (106.00 – 133.67; [105.50] – 133.67)	143.73 ± 1.73 [144.49 ± 1.62] (129.50 – 164.50; [129.50 – 164.50])	**<.001 [<.001]**
DBP (mmHg)	76.05 ± 1.27 [74.96 ± 1.28] (64.00 – 84.20; [60.75 – 84.20])	87.48 ± 0.95 [87.54 ± 0.98] (80.50 – 98.50; [76.75 – 98.50])	**<.001 [<.001]**
Screening BP			
SBP (mmHg)	123.92 ± 1.42 (112.00 – 139.50)	149.35 ± 2.15 (128.50 – 170.00)	**<.001**
DBP (mmHg)	78.25 ± 1.15 (65.00 – 89.50)	95.15 ± 1.45 (74.00 – 107.00)	**<.001**
Resting Study BP			
SBP (mmHg)	116.96 ± 2.14 (105.50 – 150.75)	138.03 ± 2.15 (120.50 – 159.25)	**<.001**
DBP (mmHg)	74.05 ± 1.56 (60.75 – 91.25)	84.54 ± 1.60 (67.75 – 97.50)	**<.001**
HR (bpm)	68.29 ± 1.60 (53.50 – 82.00)	*n* = 23 70.22 ± 1.84 (55.25 – 87.50)	.44
MAP (mmHg)	90.91 ± 1.22 (80.25 – 103.54)	107.79 ± 1.35 (97.67 – 119.88)	**<.001**

Values are means ± standard error of the mean (range); BMI, body mass index; BP, blood pressure; SBP, systolic blood pressure; DBP, diastolic blood pressure; home blood pressure with missings substituted by mean resting study BP of study days 2 and 3 is indicated in [square brackets]; MAP, mean arterial blood pressure calculated from a total of six BP measurements comprising four resting study BP measurements obtained on study days 2 and 3 and the mean of the two screening BP measurements; n = sample size; deviating sample sizes of a parameter are indicated; statistically significant results are highlighted in bold.

### 3.2 Verification of Infusion Procedures

#### 3.2.1 Verification of Effective Phentolamine Application

Successful PHE application was verified by considering the cardiovascular changes in reaction to infusion 1. Over all participants, DBP and HR changes to infusion 1 differed between the three trials (main effects of trial: DBP: *F*(2,86)=10.82, *p*<.001, *η^2^_p_* =.201,*f*=.50; HR: *F*(1.96,82.23)=4.69, *p*=.012, *η^2^_p_* =.101,*f*=.33) while changes in SBP did not (*p*=.43). As expected, *post-hoc* tests revealed that PHE immediately reduced DBP and increased HR in trial-3 (*p*’s<.001), while Sal in trial-1 and trial-2 did not (*p*’s≥.28; see **Table 2**). Notably, there were no differences between HT and NT in their reactivity to infusion 1 (interactions trial-by-group: *p*’s≥.37).

**Table 2:** Cardiovascular reactivity to infusion 1: SBP, DBP, and HR levels pre and post infusion 1.

		Trial 1 (Sal)			Trial 2 (Sal)			Trial 3 (PHE)			
		Pre-infusion 1	Post-infusion 1	*p*	Pre-infusion 1	Post-infusion 1	*p*	Pre-infusion 1	Post-infusion 1	*p*	*p*(1 vs. '2 vs. 3)
SBP (mmHg)	All (*n*=48)	*n*=47 127.90±2.24	*n*=47 127.79±2.55	n.a.	*n*=47 128.99±2.42	*n*=47 127.13±2.51	n.a.	130.41±2.56	126.08±2.28	n.a.	.43
	NT (*n*=24)	118.81±2.49	118.54±3.34	n.a.	117.79±2.37	116.46±3.01	n.a.	119.98±3.14	117.88±3.01	n.a.	*.37*
	HT (*n*=24)	*n*=23 137.39±2.59	*n*=23 137.43±2.69	n.a.	*n*=23 140.67±2.57	*n*=23 138.26±2.44	n.a.	140.83±2.75	134.29±2.50	n.a.	
DBP (mmHg)	All (*n*=48)	*n*=47 79.54±1.44	*n*=47 80.19±1.48	.28	*n*=47 80.23±1.57	*n*=47 80.36±1.44	.83	80.65±1.43	74.25±1.51	**<.001**	**<.001**
	NT (*n*=24)	74.69±1.78	75.42±1.98	.46	74.23±1.86	74.88±1.60	.44	75.94±1.80	69.13±2.19	**<.001**	*.43*
	HT (n=24)	n=23 84.59±1.76	*n*=23 85.17±1.70	.42	*n*=23 86.50±1.80	*n*=23 86.09±1.78	.64	85.35±1.78	79.38±1.49	**.001**	
HR (bpm)	All (*n*=48)	*n*=47 69.23±1.46	*n*=47 68.91±1.56	.71	*n*=47 70.70±1.35	*n*=47 71.00±1.45	.64	*n*=47 70.66±1.50	*n*=47 78.15±1.87	**<.001**	**.012**
	NT (*n*=24)	70.10±2.30	69.67±2.36	.69	70.50±2.12	70.79±2.36	.73	*n*=23 70.87±2.37	*n*=23 77.13±2.78	**<.001**	*.47*
	HT (*n*=24)	*n*=23 69.41±2.06	*n*=23 69.26±2.30	.90	*n*=23 71.97±1.91	*n*=23 72.22±1.88	.76	70.46±1.91	79.13±2.56	**<.001**	

Values are means ± standard error of the mean; SBP, systolic blood pressure; DBP, diastolic blood pressure; HR, heart rate; n, sample size; deviating sample sizes of a parameter are indicated; Sal, saline; PHE, phentolamine; n.a., not applicable; p = p-value of dependent t-tests between pre- and post-infusion 1; p (1 vs. 2 vs. 3) = p-value of comparison of infusion 1 induced changes (post-infusion 1 – pre-infusion 1) of all three experimental trials by means of repeated measures ANCOVAs with group as independent variable; interactions group-by-time are displayed in italics, main effects of time in regular upright font; statistically significant results are highlighted in bold.

#### 3.2.2 Trial Comparisons in Baseline and Infusion-Induced Changes in Plasma Catecholamines

##### 3.2.2.1 Baseline

As depicted in **Table 3**, there were no baseline (i.e. pre-infusion) differences between the three trials in plasma catecholamine levels across all participants (*p*’s≥.39). While baseline EPI levels did not differ between HT and NT (*p*=.67), HT showed higher mean NE baseline values of borderline significance (*F*(1,47)=3.28, *p*=.077, *η^2^_p_* =.076,*f*=.29).

**Table 3:** Catecholamine levels at baseline and in reaction to infusions.

		Trial 1 (Sal+Sal)	Trial 2 (Sal+NE)	Trial 3 (PHE+NE)	*p* (Trials)				*p* (HT vs. NT)
					1 vs. 2 vs. 3	1 vs. 2	1 vs. 3	2 vs. 3	1 vs. 2 vs. 3
NE baseline (pg/ml)	All (*n*=44)	427.18 ± 32.32 (187.61 – 1029.34)	426.79 ± 33.13 (145.61 – 1166.00)	427.34 ± 30.41 (126.10 – 1003.39)	.53	n.a.			*.077*
	NT (*n*=23)	385.16 ± 38.42 (190.20 – 871.22)	372.49 ± 43.20 (145.61 – 1097.49)	370.16 ± 31.12 (126.10 – 722.66)	.56	n.a.			
	HT (*n*=21)	473.19 ± 52.19 (187.61 – 1029.34)	486.25 ± 48.55 (170.21 – 1166.00)	489.95 ± 51.23 (223.35 – 1003.39)	.91	n.a.			
EPI baseline (pg/ml)	All (*n*=44)	29.33 ± 2.46 (6.00 – 66.01)	30.88 ± 2.52 (6.00 – 80.55)	29.96 ± 2.46 (6.00 – 77.96)	.39	n.a.			*.67*
	NT (*n*=23)	30.18 ± 3.74 (6.00 – 66.01)	29.08 ± 3.02 (6.00 – 58.65)	28.05 ± 2.61 (6.00 – 49.52)	.81	n.a.			
	HT (*n*=21)	28.39 ± 3.20 (6.00 – 58.88)	32.87 ± 4.15 (6.00 – 80.55)	32.05 ± 4.31 (6.00 – 77.96)	.41	n.a.			
NE change (pg/ml)	All (*n*=44)	2.99 ± 13.04 (-311.47 – 281.97)	653.59 ± 52.67 (138.55 – 1460.21)	715.90 ± 50.14 (182.45 – 1661.87)	**<.001**	**<.001**	**<.001**	.33	.53
	NT (*n*=23)	10.26 ± 17.90 (-183.35 – 281.97)	671.54 ± 71.63 (138.55 – 1232.73)	780.91 ± 77.16 (331.67 – 1661.87)	**<.001**	**<.001**	**<.001**	.20	
	HT (*n*=21)	-4.97 ± 19.32 (-311.47 – 195.35)	633.93 ± 79.24 (198.79 – 1460.21)	644.72 ± 60.54 (182.45 – 1183.28)	**<.001**	**<.001**	**<.001**	.91	
EPI change (pg/ml)	All (*n*=44)	3.83 ± 1.85 (-20.14 – 37.51)	-4.78 ± 1.07 (-27.61 – 11.20)	-0.69 ± 1.26 (-12.60 – 36.42)	.065	**<.001**	**.027**	**.005**	.39
	NT (*n*=23)	3.14 ± 2.43 (-20.14 – 37.51)	-4.48 ± 1.30 (-16.41 – 11.20)	0.88 ± 2.09 (-10.23 – 36.42)	.089	**.003**	.38	**.009**	
	HT (*n*=21)	4.59 ± 2.87 (-13.34 – 35.10)	-5.11 ± 1.77 (-27.61 – 8.22)	-2.41 ± 1.29 (-12.60 – 8.97)	.51	**.010**	**.035**	.21	

Values are means ± standard error of the mean; NE, norepinephrine; EPI, epinephrine; NT, normotensive participants; HT, hypertensive participants; n, sample size; Sal, saline; PHE, phentolamine; n.a., not applicable; p (Trials), p-values of comparisons of in EPI or NE (1 minute post second infusion – baseline measurements) of all three trials by means of repeated measures ANCOVAs with group (HT vs. NT) as independent variable and trial order as covariate (1 vs. 2 vs. 3) or by means of two trial comparisons by means of dependant t-tests (1 vs. 2; 1 vs. 3; 2 vs. 3) for all participants and for HT and NT separately; p (HT vs. NT) = p-values of group comparisons of baseline levels or infusion-induced changes by means of repeated measures ANCOVAs with group (HT vs. NT) as independent variable and trial order as covariate, interactions group-by-trial are displayed in regular upright font, main effects of group in italics; statistically significant results are highlighted in bold.

##### 3.2.2.2 Trial Comparisons

Infusions of the three trials induced differential catecholamine changes (main effects trial: NE: *F*(2,80)=43.80, *p*<.001, *η^2^_p_* =.523,*f*=1.05; EPI: *F*(2,80)=2.83, *p*=.065, *η^2^_p_* =.066,*f*=.27) as follows: *NE.* As compared to Sal-only infusion, Sal+NE and PHE+NE led to increased NE plasma levels as expected (*p*´s<.001) without difference between Sal+NE and PHE+NE (*p*=.33) (see **Table 3**). Within trial comparisons revealed NE increases after Sal+NE and PHE+NE (*p*´s<.001), but not after Sal+Sal (*p*=.82). *EPI.* As compared to Sal+Sal, Sal+NE and PHE+NE led to significant decreases in EPI levels (*p*´s≤.027) with smaller decreases after PHE+NE (−0.69 pg/ml) as compared to Sal+NE (−4.78 pg/ml; *t*(43)=−2.93, *p*=.005). Within trial comparisons revealed that Sal-only infusion increased EPI levels (Sal+Sal: *t*(43)=−2.07, *p*=.044) while NE-infusion decreased EPI levels (Sal+NE: *t*(43)=4.46, *p*<.001) but not with prior PHE-infusion (PHE+NE: *p*=.58).

Infusion-induced catecholamine changes did not differ between hypertensive participants and normotensives controls (main effect group: *p*’s≥.23; interactions trial-by-group: *p*’s≥.39).

### 3.3 Cardiovascular Reactivity to NE-Infusion Without and With Alpha-Adrenergic Blockade in HT and NT

#### 3.3.1 Systolic Blood Pressure

HT and NT significantly differed in their SBP reactivity across the three experimental trials, as is indicated by the significant interaction trials-by-group-by-time (*F*(9.83,422.82)=2.93, *p*=.002, *η^2^_p_* =.064,*f*=.26). We next calculated *post-hoc* comparisons between two trials (i.e. Sal+Sal vs. Sal+NE; Sal+Sal vs. PHE+NE; Sal+NE vs. PHE+NE). Compared to Sal-only infusion, both, Sal+NE and PHE+NE induced higher SBP reactivity in HT as compared to NT (interactions trials-by-group-by-time: Sal+Sal vs. Sal+NE: *F*(5,215)=2.48, *p*=.033, *η^2^_p_* =.055,*f*=.24; Sal+Sal vs. PHE+NE: *F*(4.79,205.93)=2.80, *p*=.015, *η^2^_p_* =.064,*f*=.26). Moreover, SBP reactivity to NE-infusion without and with α-AR blockade significantly differed between the two groups [interaction trials-by-group-by-time: Sal+NE vs. PHE+NE: *F*(5,215)=3.30, *p*=.007, *η^2^_p_* =.071,*f*=.28]. While HT showed similar reactivity to NE-infusion without and with prior α-AR blockade, the reactivity of NT to NE-infusion with α-AR blockade was attenuated, rather resembling their reactivity to Sal-only infusion. Finally, separate analyses of each trial alone revealed greater SBP increases in HT as compared to NT after Sal+NE and PHE+NE, but not after Sal+Sal, independent of age and BMI (interactions group-by-time: Sal+Sal: *p*=.21; Sal+NE: *F*(5,205)=2.40, *p*=.038, *η^2^_p_* =.055,*f*=0.24; PHE+NE: *F*(5,205)=2.85, *p*=.016, *η^2^_p_* =.065,*f*=0.26).

Complementary analyses with MAP as continuous independent variable instead of group confirmed these results: SBP reactivity differed across all three trials with increasing MAP (interaction trials-by-MAP-by-time: *F*(9.82,422.32)=3.98, *p*<.001, *η^2^_p_* =.085,*f*=.30). While SBP reactivity did not change with increasing MAP after Sal+Sal, SBP reactivity was highest with increasing MAP after Sal+NE followed by PHE+NE with Sal+NE and PHE+NE significantly differing from each other. Two-trial comparisons revealed differences between all trials (interactions trials-by-MAP-by-time: Sal+Sal vs. Sal+NE: *F*(5,215)=3.01, *p*=.012, *η^2^_p_* =.065,*f*=.26; Sal+Sal vs. PHE+NE: *F*(5,215)=5.31, *p*<.001, *η^2^_p_* =.110,*f=*.35; Sal+NE vs. PHE+NE: *F*(5,215)=3.79, *p*=.003, *η^2^_p_* =.081,*f=*.30). Separate analyses of each trial alone confirmed SBP increases with increasing MAP after Sal+NE and PHE+NE but not Sal+Sal independent of age and BMI (interactions MAP-by-time: Sal+Sal: *p*=.23, Sal+NE: *F*(5,205)=2.88, *p*=.016, *η^2^_p_* =.066,*f*=.27; PHE+NE: *F*(5,205)=3.97, *p*=.002, *η^2^_p_* =.088,*f*=.31).

**Figure 3:**
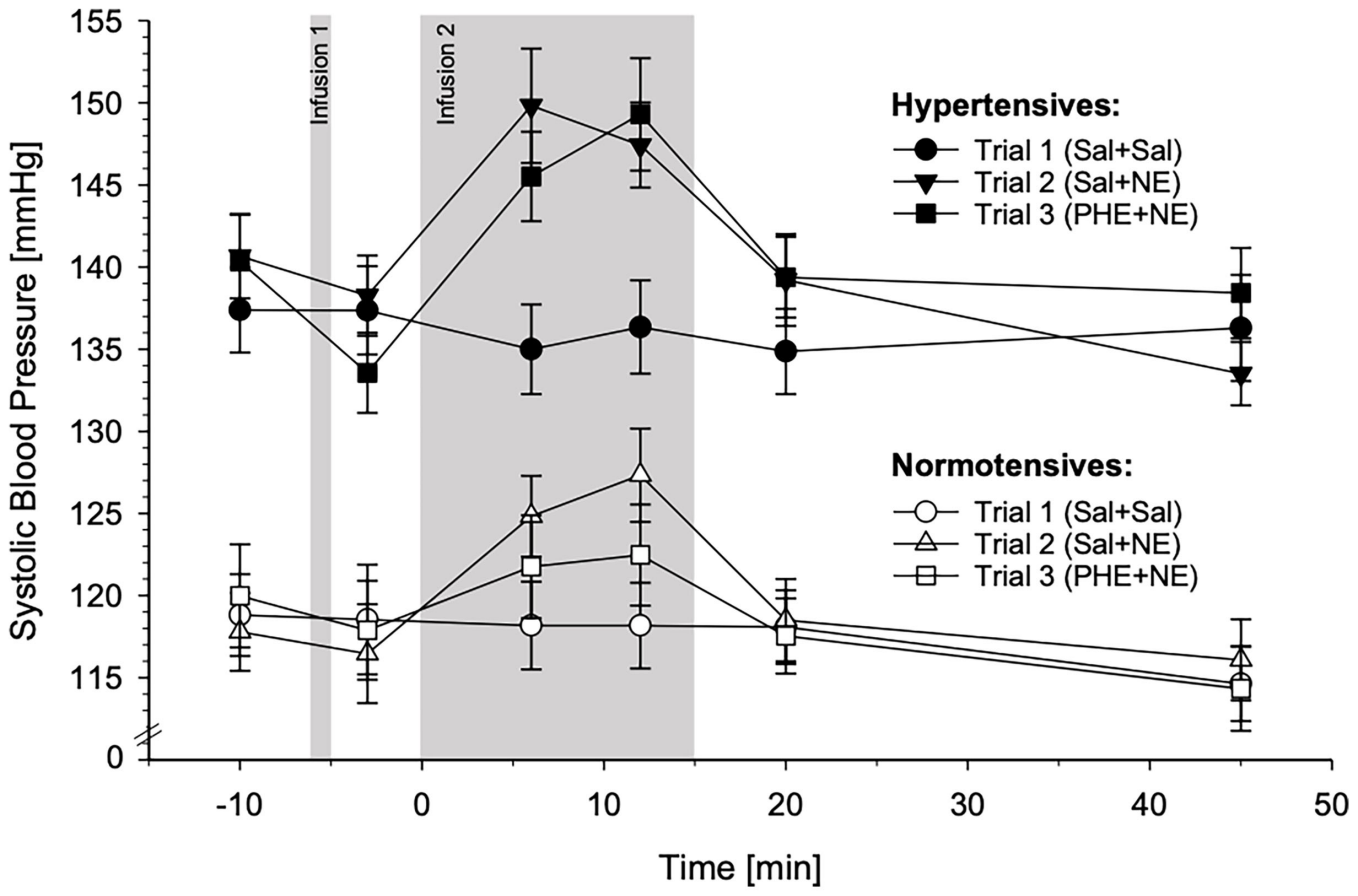
Systolic blood pressure (SBP) reactivity to the three different substance infusion-trials (trial 1: Sal+Sal, circles; trial 2: Sal+NE, triangles; trial 3: PHE+NE, rectangles) in hypertensive participants (HT; black symbols; *n* = 23) and normotensive controls (NT; white symbols; *n* = 24) (*mean* ± *SEM*). SBP reactivity differed across the three trials between HT and NT as revealed by the calculated general linear model (interaction trials-by-group-by-time: *p* = .002). *Pairwise trial comparisons by means of general linear models*: In comparison to NT, HT displayed higher SBP reactivity to Sal+NE as compared to Sal+Sal (*p* = .033). Moreover, HT and NT differed in their SBP reactivity to PHE+NE as compared to Sal+Sal (*p* = .015) and to Sal+NE (*p* = .007): In NT, SBP reactivity to PHE+NE was markedly reduced as compared to Sal+NE, whereas HT showed a similar reactivity to Sal+NE and PHE+NE. *Within each trial by means of repeated measures ANCOVAs*: HT showed higher SBP reactivity to Sal+NE and PHE+NE (*p’s* ≤.038) but not to Sal+Sal (*p* = .21).

SBP reactivity in response to the three different substance infusion trials for HT and NT is depicted in **Figure 3**. For graphical illustrations of SBP reactivity to the different trials separately, please refer to **Supplementary Figure 1**.

#### 3.3.2 Diastolic Blood Pressure

DBP reactivity also differed significantly between HT and NT across the three experimental trials, as is indicated by the significant interaction trials-by-group-by-time (*F*(9.28,399.09)=2.33, *p*=.014, *η^2^_p_* =.051,*f*=.23). Here, *post-hoc* comparisons between two trials revealed that as compared to Sal-only infusion, NE-infusion without α-AR blockade induced higher DBP reactivity in HT than in NT (interactions trials-by-group-by-time: Sal+Sal vs. Sal+NE: *F*(4.55,195.63)=3.40, *p*=.007, *η^2^_p_* =.073,*f*=.28). This group difference disappeared with α-AR blockade: HT and NT differed in their DBP reactivity between NE-infusion with and without α-AR blockade (interactions trials-by-group-by-time: Sal+Sal vs. PHE+NE: *p*=.55; Sal+NE vs. PHE+NE: *F*(5,215)=2.66, *p*=.024, *η^2^_p_* =.058,*f*=.25). Further separate *post-hoc* analyses of the trials confirmed enhanced DBP reactivity in HT after NE-infusion without blockade but not with prior PHE application independent of age and BMI (interactions group-by-time: Sal+Sal: *p*=.29; Sal+NE: *F*(4.90,200.98)=2.48, *p*=.034, *η^2^_p_* =.057,*f*=.25; PHE+NE: *p*=.26).

Complementary analyses using MAP as the linear independent variable instead of group confirmed the above reported difference in DBP reactivity across the three experimental trials dependent on MAP (interactions trials-by-MAP-by-time: *F*(9.53,409.80)=2.96, *p*=.002, *η^2^_p_* =.064,*f*=.26). Moreover, *post-hoc* comparisons between two trials with MAP as linear independent variable instead of group revealed higher DBP reactivity with increasing MAP following Sal+NE as compared to Sal+Sal but not as compared to PHE+NE (interactions trials-by-group-by-time: Sal+Sal vs. Sal+NE: *F*(4.70,202.10)=4.52, *p*=.001, *η^2^_p_* =.095, *f*=.32; Sal+Sal vs. PHE+NE: *p*=.25; Sal+NE vs. PHE+NE: *F*(5,215)=2.86, *p*=.016, *η^2^_p_* =.062,*f*=.26), similar to the analyses with group as the independent variable. Again, further *post-hoc* analyses of the separate trials with MAP as the linear independent variable instead of group confirmed higher DBP reactivity with increasing MAP after Sal+NE but not PHE+NE or Sal+Sal independent of age and BMI (interactions group-by-time: Sal+Sal: *p*=.14; Sal+NE: *F*(5.00,205.00)=3.67, *p*=.003, *η^2^_p_* =.082,*f*=.30; PHE+NE: *p*=.39).

**Figure 4:**
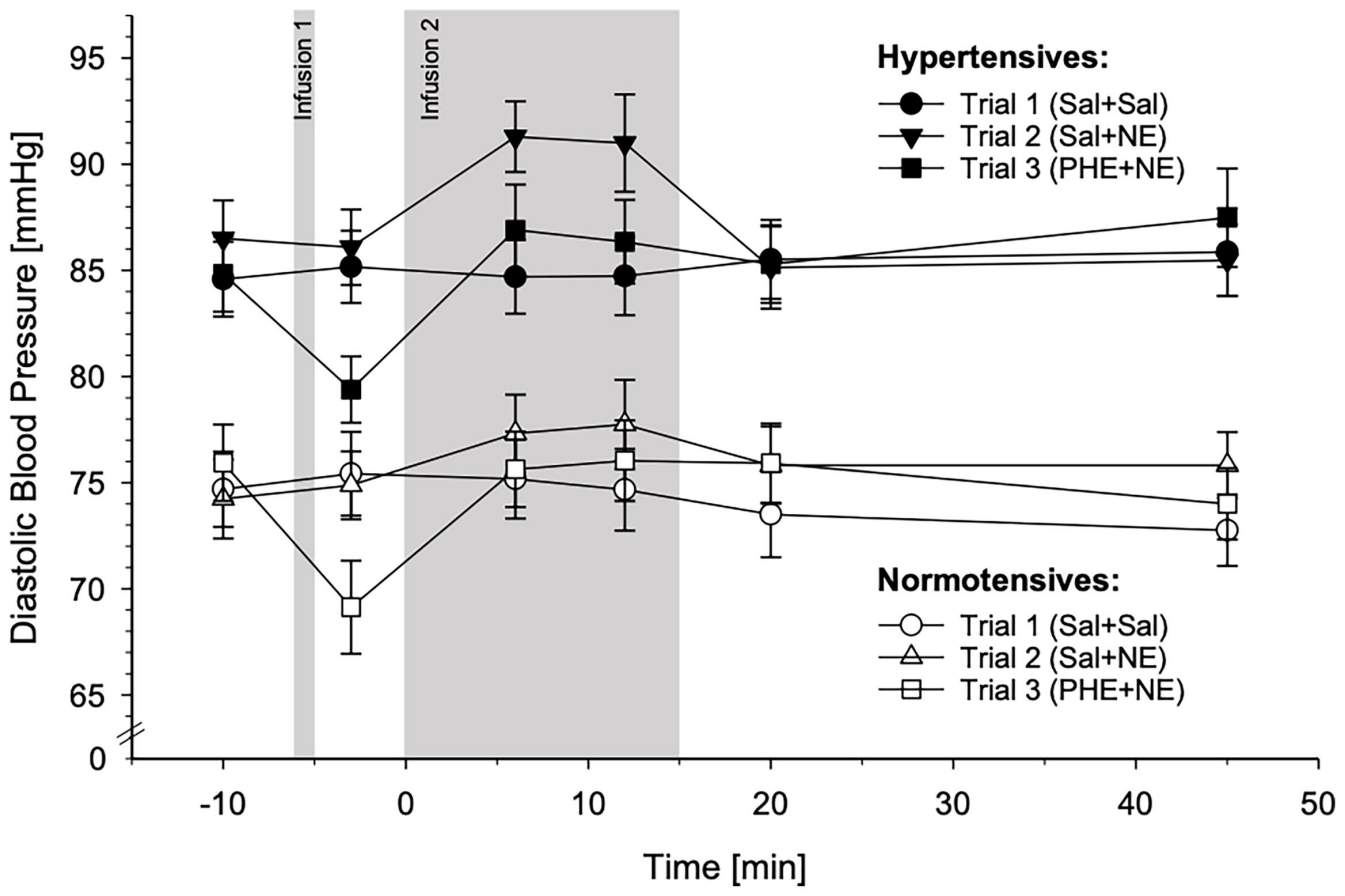
Diastolic blood pressure (DBP) reactivity to the three different substance infusion-trials (trial 1: Sal+Sal, circles; trial 2: Sal+NE, triangles; trial 3: PHE+NE, rectangles) in hypertensive participants (HT; black symbols; *n* = 23) and normotensive controls (NT; white symbols; *n* = 24) (*mean* ± *SEM*). DBP reactivity differed across the three trials between HT and NT as revealed by the calculated general linear model (interaction trials-by-group-by-time: *p* = .014). *Pairwise trial comparisons by means of general linear models*: In comparison to NT, HT displayed higher DBP reactivity to Sal+NE as compared to Sal+Sal (*p* = .007). PHE-induced α-adrenergic receptor blockade dampened reactivity to NE in both, HT and NT, resulting in similar reactivity to Sal+Sal and PHE+NE (Sal+Sal vs. PHE+NE: *p* = .55; Sal+NE vs. PHE+NE: *p* = .024). *Within each trial by means of repeated measures ANCOVAs*: DBP reactivity of HT and NT to did not differ to Sal+Sal and PHE+NE (*p*’s ≥.26) but to Sal+NE where HT exhibited higher reactivity as compared to NT (*p* = .034).

**Figure 4** depicts DBP reactivity in response to the three different substance infusion trials for HT and NT. For graphical illustrations of DBP reactivity to the different trials separately, please refer to **Supplementary Figure 2**.

#### 3.3.3 Heart Rate

HT and NT did not differ in their HR reactivity across the three experimental trials, either using group as independent variable or MAP (interactions trials-by-group-by-time: *p*’s≥.73).

**Figure 5:**
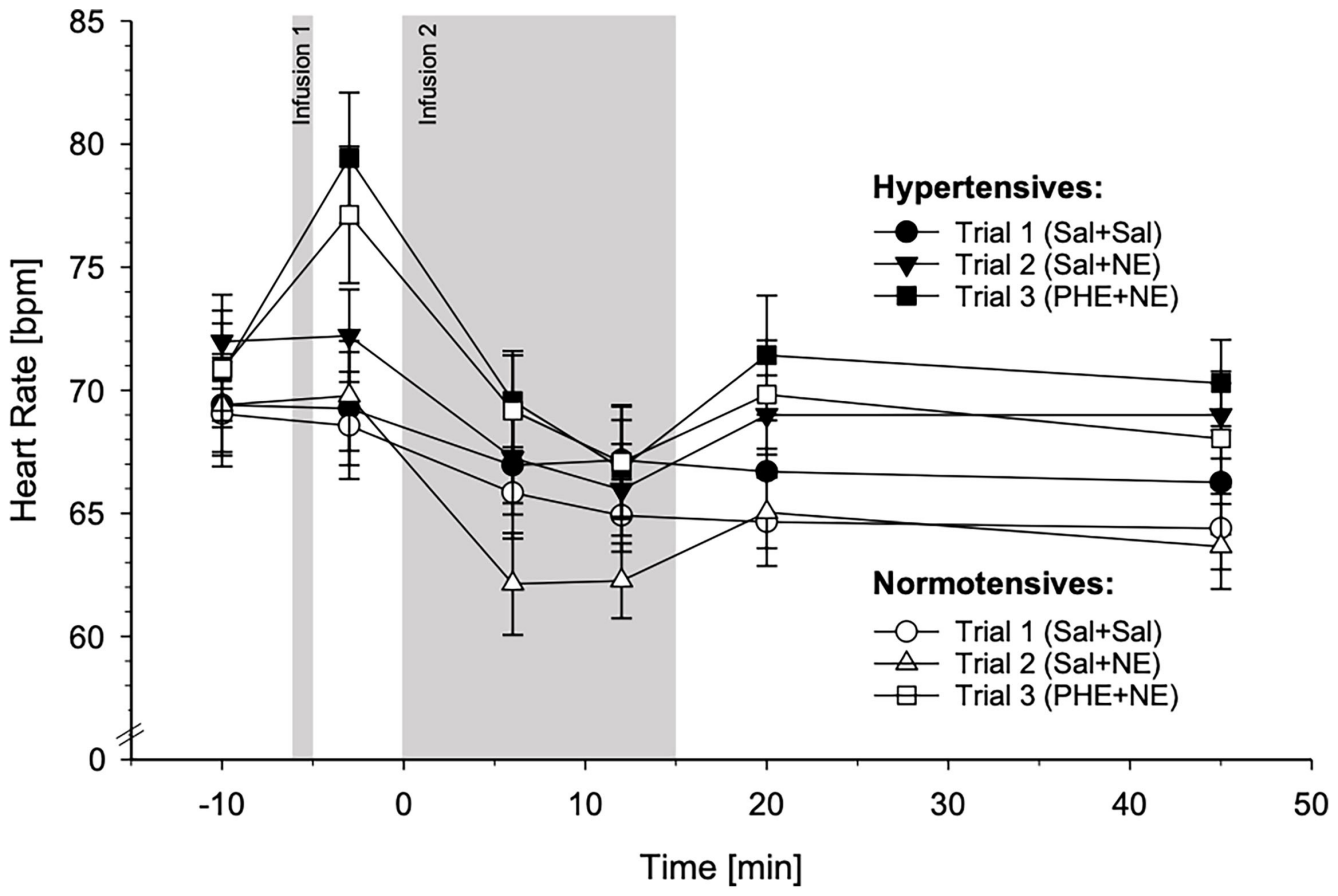
Heart rate (HR) reactivity to the three different substance infusion-trials (trial 1: Sal+Sal, circles; trial 2: Sal+NE, triangles; trial 3: PHE+NE, rectangles) in hypertensive participants (HT; black symbols; *n* = 23) and normotensive controls (NT; white symbols; *n* = 23) (*mean* ± *SEM*). HR reactivity across the three trials did not differ between HT and NT as revealed by the calculated general linear model (interaction trials-by-group-by-time: *p* = .73).

HR reactivity in response to the three different substance infusion trials for HT and NT is depicted in **Figure 5**. For graphical illustrations of HR reactivity to the different trials separately, see **Supplementary Figure 3**.

## 4 Discussion

Here, we investigated the role of α-AR mechanisms in the cardiovascular hyperreactivity to a NE-stress reactivity-mimicking NE-infusion in EHT as compared to NT. We repeatedly assessed BP and HR before, during, and after standardized NE-infusion with and without prior non-selective α-AR blockade by PHE as well as before, during, and after placebo Sal-infusions.

The *first main finding* of our study is that we observed hyperreactivity of SBP and DBP to the same NE-infusion in EHT as compared to NT. The observed greater BP reactivity in HT as compared to NT is in line with previous studies that found increased cardiovascular responsiveness or reactivity to a vasoconstrictor stimulus such as NE in HT (10, 11). In contrast to these previous studies, we infused a standardized, not weight-adjusted dose of NE. Thus, our study provides further evidence for BP hyperreactivity to a given NE-infusion in EHT applying a more conservative methodology given the generally higher body weight of HT as compared to NT (2).

With respect to underlying mechanisms, our *second main finding* was that the observed DBP hyperreactivity but not SBP hyperreactivity in EHT was attenuated after prior non-selective α-AR blockade by PHE. More precisely, *DBP reactivity* to NE-infusion with prior α-AR blockade was comparable between HT and NT (except for the overall higher DBP levels in HT) and similar to their reactivity to Sal-infusion with both, HT and NT, showing little to no reactivity. In contrast, *SBP reactivity* to NE-infusion with prior α-AR blockade differed between HT and NT. While HT exhibited similar SBP reactivity to NE-infusion without and with prior α-AR blockade, SBP reactivity of NT to NE-infusion with α-AR blockade was attenuated. To sum up, our results indicate that DBP hyperreactivity, but not SBP hyperreactivity to NE-infusion, can be almost completely inhibited by α-AR blockade in EHT. This points to a modulating role of α-AR in the DBP hyperreactivity but not in the SBP hyperreactivity to NE observed in EHT.

Our *DBP results* are in line with previous literature in either HT or NT. In HT, higher NE dosages were required to induce DBP-increases of 20 mmHg after α_1_-AR blockade (25) and MAP reactivity to NE-infusion was attenuated after α_1_-AR blockade (24), both as compared to placebo. Notably, DBP has a stronger impact on calculated MAP (MAP=2/3 DBP+1/3 SBP) than SBP. Similarly, DBP reactivity to mild mental stress was attenuated in EHT after α_1_-AR blockade as compared to reactivity under placebo conditions (45). In NT, the observed blunted DBP reactivity to NE-infusion after non-selective α-AR blockade is consistent with earlier NT studies on DBP reactivity to NE-infusion after selective α_1_-, selective α_2_-, and non-selective α-AR blockade (22, 23). Attenuation of DBP reactivity in HT and NT after non-selective α-AR blockade likely results from inhibition of α_1_- and α_2_-AR activation-induced vasoconstriction of vascular smooth muscles by PHE as peripheral vascular tone and resistance determine DBP (46, 47). Given that the DBP hyperreactivity to NE-infusion observed in our HT was eliminated by prior α-AR blockade by PHE resulting in similar DBP reactivity to NE-infusion after α-AR blockade in HT and NT, it can be assumed that the DBP hyperreactivity to NE in EHT is mediated *via* α_1_- and α_2_-AR. More specifically, we speculate that functional alterations in α_1_- and α_2_-AR located in vascular smooth muscle cells predominantly account for the DBP hyperreactivity to NE in EHT based on the role of vascular tone in DBP (47, 48). Whether these supposed functional alterations of vascular α_1_- and α_2_-AR result from an increased number (49) or a hypersensitization of α-AR (50) in HT compared to NT has not yet been studied. So far, α_2_-AR density and responsiveness in human EHT have been investigated in platelets from peripheral blood. Notably, platelets do express α_2_- but not α_1_-AR whereas vascular smooth muscle cells do express both (47, 51). While results are inconclusive regarding platelet α_2_-AR density (52), sensitivity of platelet α_2_-AR to adrenergic stimulation was found to be enhanced (53). It is unclear whether these α_2_-AR results obtained in platelets may also apply to vascular smooth muscle cells and α_1_-AR (54).

With respect to *SBP*, our finding that α-AR blockade did not affect SBP reactivity to NE-infusion in HT expands the hitherto available body of knowledge as SBP alone has not been considered in the context of NE-infusion with α-AR blockade in HT so far. One study investigated cardiovascular reactivity to mild mental stress after chronic α_1_-AR blockade but found attenuated SBP reactivity in EHT (45) which is contrary to our results. An explanation for this divergency might be that we used acute blockade while that study used chronic blockade. Notably, chronic blockade can cause myocardial remodeling with effects on BP reactivity (55). The observed attenuated SBP reactivity to NE-infusion after non-selective α-AR blockade in our NT is consistent with earlier NT studies considering reactivity to NE-infusion after selective α_1_-, selective α_2_-, and non-selective α-AR blockade (22, 23). In NT, α-AR contribute to SBP-increases in reaction to NE-infusion primarily by the activation of α_1_-AR in cardiomyocytes that induce increased contractile force of the left ventricle but also by activation of vascular α_1_- and α_2_-AR that increases venous return and thus left ventricular preload (56). Thus, in NT, α-AR blockade likely prevents these effects and consequently reduces SBP reactivity to NE-infusion. Notably, cardiac β-AR also contribute to ventricular contractility (15) which might explain why SBP reactivity to NE-infusion after α-AR blockade was reduced and not abolished in NT. In contrast, we observed exaggerated SBP reactivity to NE-infusion with and without α-AR blockade in our HT. In light of the reasoning above and our DBP results, our SBP findings suggest functional alterations in terms of reduced functionality of α-AR, supposedly in cardiomyocytes, in EHT, either by desensitization or reduced expression density. Alternatively, the remaining SBP-hyperreactivity to NE-infusion with prior α-AR blockade may result from reflex-induced sympathetic activation of β-AR due to the BP-reduction by PHE which was more pronounced (not statistically significant) in HT (57). However, HR which is regulated predominantly by β-AR did not increase following NE infusion and HT and NT did not differ in their HR reactivity. Further explanations may relate to potential vascular remodeling leading to generalized increases in vascular reactivity to constrictor stimuli (58), or increased stroke volume and cardiac output following NE-infusion (59, 60).

We interpret our SBP and DBP findings in that they point to a divergency in α-AR functioning between cardiomyocytes and vascular smooth muscle cells in EHT with increased functioning in vascular cells and decreased functioning in cardiomyocytes. As α-AR functioning does not seem to explain the observed SBP hyperreactivity in EHT, the underlying mechanisms remain to be elucidated in future studies.

With respect to *HR reactivity*, we did not observe reactivity differences between HT and NT to any of the infusions. In reaction to high-dose NE-infusion as applied in this study, HT and NT exhibited similar HR reactivity or bradycardia, respectively, in our study and in earlier studies (61, 62). The NE-infusion-induced bradycardia as well as the PHE-infusion-induced tachycardia are supposedly mediated by the baroreceptor reflex (15, 63). Given the similar HR reactivity in HT and NT but at the same time more pronounced BP changes to both infusions in HT (not statistically significant for PHE), our results further support the impairment in baroreceptor reflex functioning in EHT (64). HR reactivity to NE-infusion with prior α-AR blockade by PHE did not differ between HT and NT and resembled their reactivity to NE-infusion without α-AR blockade. Our results are in line with earlier studies considering HR reactivity to NE-infusion either in NT after selective α_1_-, selective α_2_-, or non-selective α-AR blockade (22, 23) or in HT after α_1_-AR blockade (24), all as compared to placebo. Similarly, HR reactivity to mental stress was not reduced after α_1_-AR blockade in HT (45) and in NT (65). Not surprisingly, α-AR blockade did not affect HR reactivity to NE as HR reactivity is mediated *via* β-AR (15).

Our findings may have *clinical implications*. First, given the supposed functional alterations of α-AR underlying the observed DBP hyperreactivity to NE in EHT, the use of α-AR blockers as add-on in the antihypertensive treatment might be beneficial under certain circumstances, e.g. in HT with prostate related symptoms (66), metabolic complications (67), and/or inadequate responsiveness to standard medication (68). Second, the inhibition-resistance of SBP reactivity in EHT may point to a mechanism involved in development and maintenance of EHT. More precisely, with reference to the allostatic load model (69), SBP hyperreactivity may add to “repeated hits” as type of allostatic load that accumulates over time to chronic elevation of BP and thus hypertension.

*Strengths* of our study include the application of a placebo-controlled within-subject Latin Square Design with a NE-infusion protocol that mimics duration and effectiveness (in terms of effects on BP) of NE-release in reaction to acute psychosocial stress by means of the TSST. Second, we administered standardized NE- and PHE-infusion dosages, what poses another strength as potential confounding of weight-adjusted infusion dosage due to the higher weight in EHT is excluded. Third, the chosen measurement timepoints allowed, in addition to the comprehensive investigation of the cardiovascular reactivity to NE-infusion with and without prior non-selective α-AR blockade, the verification of successful PHE application. Notably, we observed the known effects of PHE, namely decreases in DBP and increases in HR [e.g (70)]. Fourth, we performed a comprehensive hypertension assessment procedure including home and study BP measurements in all eligible participants. Finally, we controlled for a variety of potential confounders, both, during recruitment and in our statistical analyses. Also, our methodological approach aimed to minimize potential confounding effects on reactivity and recovery kinetics [e.g. infusion dosage (37), BMI (71), age (43), gender (28–30), activity during recovery phase (72), etc.]. Our study also has *limitations*. First, the generalizability of our findings is restricted to normotensive and hypertensive but otherwise healthy and medication-free men. Notably, further variables such as gender, race, sodium diet, physical activity, sleep, or perceived experience with substance infusion, may influence cardiovascular (NE-infusion-) reactivity, and therefore our results may vary in other populations, in particular women, even if they are healthy and medication-free (29, 30, 43, 73–76). Future studies are needed to investigate potential confounding influences of these variables on the observed reactivity differences between HT and NT. Second, our study design only allowed to specifically investigate the role of α-AR in the cardiovascular (hyper)reactivity to NE in EHT, but without combined confirmation of previous β-AR findings. Third, we did not assess further assessable hemodynamic parameters apart from BP and HR such as stroke volume or cardiac output. To investigate the mechanisms underlying the observed reactivity difference between SBP and DBP in hypertension, future studies are needed that evaluate adrenergic effects on parameters reflecting cardiac and vascular functioning. Fourth, in line with the literature our HT had a higher BMI as compared to their normotensive controls (77) which apart from the elevated BP may add to the cardiovascular risk in HT (78). However, the present study is a mechanistic study and we controlled for BMI in our statistical analyses following previous methods and scientific practice [e.g (79–81)]. Fifth, intravenous infusion of exogenous NE may have different effects on α-AR compared to endogenous NE released in reaction to mental stress (82). Sixth, we cannot rule out that the short-term effects of PHE-infusion, namely BP decreases and HR increases, affected our results. We consider it unlikely that acute baroreceptor resetting affects reactivity to the second infusion as the one min PHE infusion was too short to have such an effect (83, 84). In addition, chronic baroreceptor resetting as apparent in EHT has not been shown to alter acute baroreceptor resetting and consequently acute baroreceptor resetting would equally affect both, HT and NT (85). Seventh, given the homeostatic properties of the baroreflex (86), one could argue that the impaired baroreflex functioning in EHT (64) affected cardiovascular recovery in HT. However, our results do not suggest recovery differences between HT and NT and other research has shown that the onset of the antihypertensive response to stimulation does not seem to be altered with hypertension (87). Therefore, the baroreflex does not seem to play a major role in mediating the observed group differences in BP reactivity. Last, as we used non-selective α-AR blockade, the individual contribution of α_1_- and α_2_-AR to DBP hyperreactivity in EHT remains unclear. Given that the α_1_-AR subtype is the predominant AR in vascular smooth muscles (88), a more prominent role of α_1_-AR as compared to α_2_-AR can be assumed.

Taken together, our study sheds further light on the hitherto only sparsely known role of α-adrenergic mechanisms underlying the cardiovascular hyperreactivity to stimulation observed in EHT. We found that DBP hyperreactivity to a standardized NE-stress reactivity-mimicking NE-infusion in EHT was attenuated after prior non-selective α-AR blockade by PHE while SBP hyperreactivity was not. This suggests on the one hand that DBP hyperreactivity to NE in EHT is mediated *via* α-AR. On the other hand, our results indicate that the functioning of α-AR involved in regulating the SBP reactivity to stimulation seems impaired in EHT. Thus, in contrast to DBP hyperreactivity, SBP hyperreactivity in EHT is unlikely to be mediated by α-AR. Future studies are needed to investigate the mechanisms underlying the SBP hyperreactivity to NE in EHT as well as clinical implications and generalizability of our findings.

## Data Availability Statement

The raw data supporting the conclusions of this article will be made available by the authors, without undue reservation.

## Ethics Statement

The studies involving human participants were reviewed and approved by Ethics Committee of the State of Bern, Switzerland and the Swiss Agency for Therapeutic Products. The participants provided their written informed consent to participate in this study.

## Author Contributions

L-MW: wrote the paper, analyzed and interpreted data, approved the manuscript to be published, agrees to be accountable for all aspects of the work in ensuring that questions related to the accuracy or integrity of the work are appropriately investigated and resolved. RK: contributed to the design of the study, contributed to the management of data acquisition, revised the manuscript for important intellectual content; approved the manuscript to be published. NH: contributed to data acquisition; revised the manuscript for important intellectual content; approved the manuscript to be published. CZ-H: contributed to data acquisition; revised the manuscript for important intellectual content; approved the manuscript to be published. GS: contributed to data acquisition; revised the manuscript for important intellectual content; approved the manuscript to be published. PW: wrote the paper; designed the study, managed data acquisition, interpreted data; approved the manuscript to be published; agrees to be accountable for all aspects of the work in ensuring that questions related to the accuracy or integrity of the work are appropriately investigated and resolved.

## Funding

This study was supported by research grants from the Swiss National Science Foundation [320030_122406, PP00P1_128565/1] and from the German Research Foundation [INST 38/550-1] (all to PHW) as well as from the German Research Foundation under Germany’s Excellence Strategy [EXC 2117—422037984]. The funding sources had no impact on study design, data collection and analysis, writing of the manuscript, or the decision to submit the manuscript for publication.

## Conflict of Interest

The authors declare that the research was conducted in the absence of any commercial or financial relationships that could be construed as a potential conflict of interest.

## Publisher’s Note

All claims expressed in this article are solely those of the authors and do not necessarily represent those of their affiliated organizations, or those of the publisher, the editors and the reviewers. Any product that may be evaluated in this article, or claim that may be made by its manufacturer, is not guaranteed or endorsed by the publisher.
