# Supplementary material for: Alpha-Adrenergic Mechanisms in the Cardiovascular Hyperreactivity to Norepinephrine-Infusion in Essential Hypertension
Source: Front Endocrinol (Lausanne). 2022 Jul 22;13:824616. doi: 10.3389/fendo.2022.824616 (PMC9355707; doi:10.3389/fendo.2022.824616)
Supplement: Supplementary file 1 [file DataSheet_1.pdf]

Supplementary Material

1 Safety aspects of infusion procedure

1.1 Physiological measures

All infusion substances and dosages are commonly used within the scope of standard treatments in different contexts. Potential side effects of NE-infusion include anxiety, general discomfort, headache, breathing difficulties, nausea, angina pectoris, renal and splanchnic ischemia, and increase in myocardial O_2 consumption (1) which usually occur in the case of high infusion dosages or too rapid infusion (2). Potential side effects of PHE include headache, restlessness and palpitation, nausea, rhinitis, diarrhea, and dizziness (3, 4).

No adverse side effects of the infusion procedures were generally expected as we recruited normotensive and hypertensive but otherwise healthy men. Both, the Swiss Agency for Therapeutic Products (Swissmedic) and the Ethical Committee of the Canton of Bern, Switzerland approved our study protocol and safety regulations. Safety regulations included that the infusion procure was performed under constant surveillance of a board-certified internist in order to be able to immediately handle unexpected medical emergencies or side effects of NE and/or PHE infusions. BP and HR were monitored continuously and in case of BP ≥ 200 mmHg systolic BP and/or 115 mmHg diastolic BP and/or HR 200 beats per minute minus participant's age, NE-infusion would have been stopped immediately. Moreover, PHE was infused in supine position to prevent reactive changes in autonomic functioning in response to the drop in BP in reaction to α-adrenergic receptor blockade by PHE. Overall, participants reported, if any, minor side effects.

1.2 Psychological measures

We cannot exclude that the administered substances are experienced differently and that participants draw conclusions from these experiences. Notably, participants were informed about the substances administered in the participant information document prior to participation. While all participants were treated equally, we cannot rule out that the participants' individual experience with infusion of the different substances may differ. Therefore, we assessed participants' emotional and physical experience, i.e. fear, physical uneasiness, and need to leave the situation, on visual analogue scales after the first infusion (Sal or PHE) and during the second infusion (Sal or NE). There were no differences between trials with respect to self-reported fear (p's $\geq .32$), physical uneasiness (p's $\geq .43$), or need to leave the situation (p's $\geq .26$) after infusion 1 (Sal or PHE) or during infusion 2 (Sal or NE).

2 Supplementary Figures and Tables

2.1 Supplementary Figures

Supplementary Figure 1

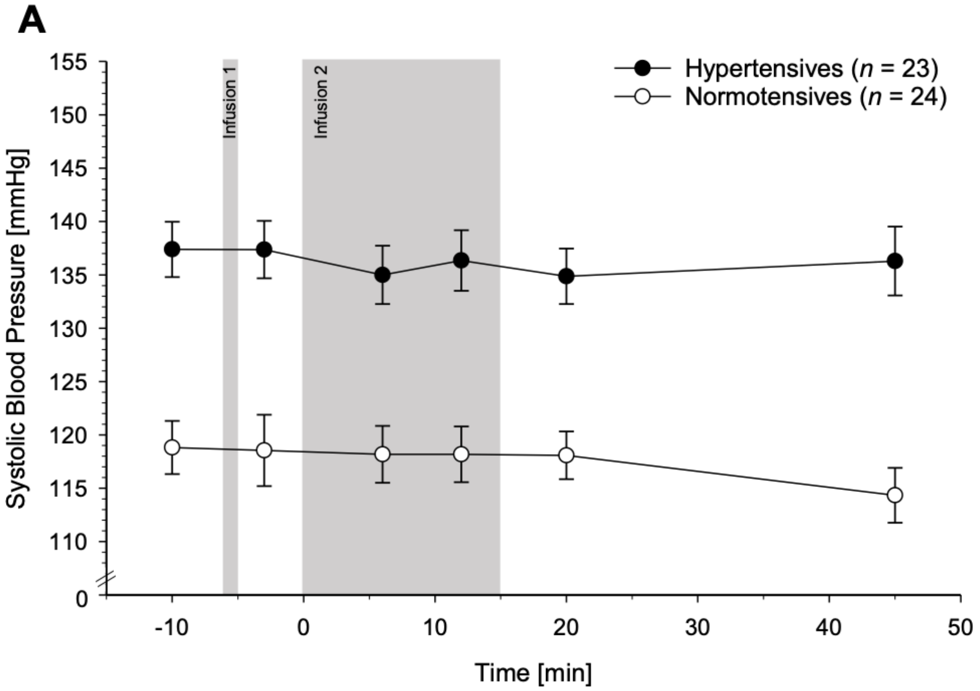

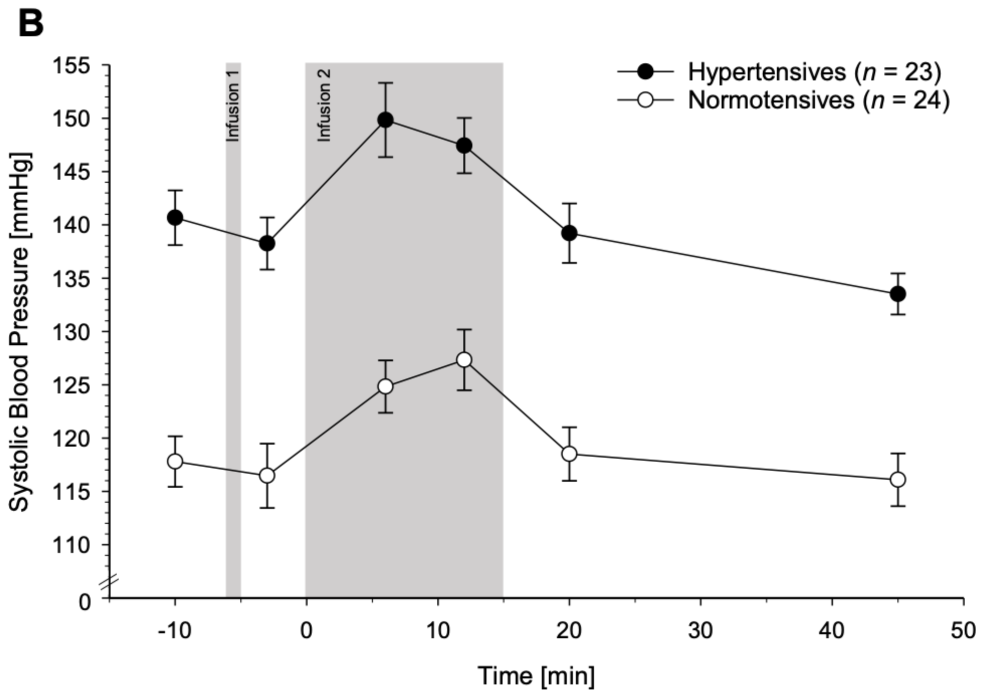

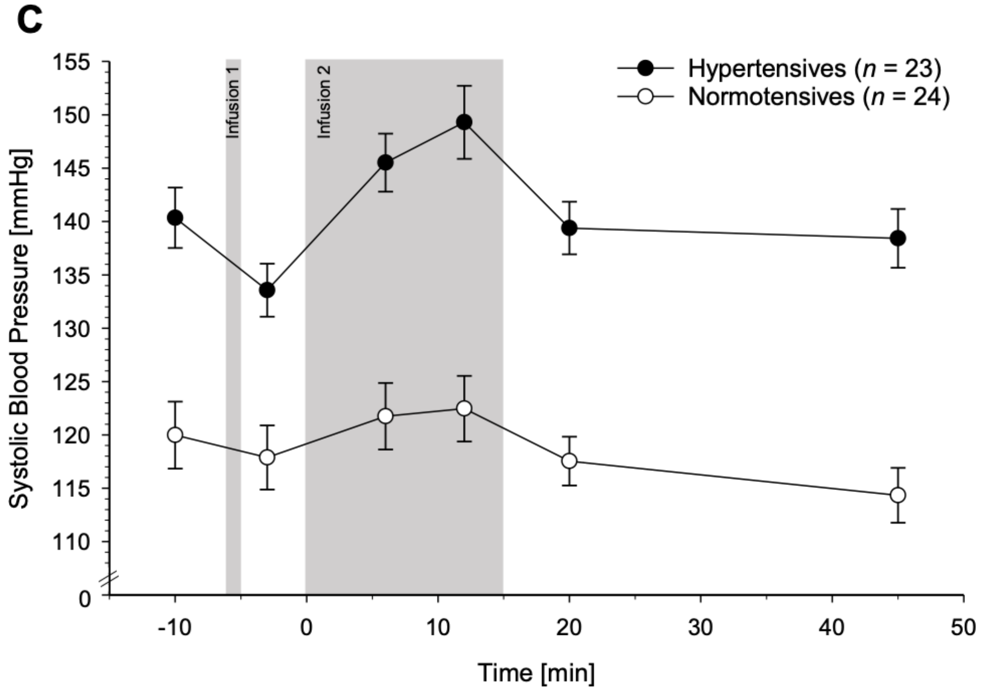

Supplementary Figure 1. Systolic blood pressure reactivity of hypertensive participants (n = 23) and normotensive controls (n = 24) to the three different substance infusions separately for each trial (mean ± *SEM*). (A) *Trial 1*: saline + saline (repeated measures ANCOVA, interaction group-by-time: p = .21). (B) *Trial 2*: saline + norepinephrine (repeated measures ANCOVA, interaction group-by-time: p = .038). (C) *Trial 3*: non-selective α-adrenergic receptor blocker

phentolamine + norepinephrine (repeated measures ANCOVA, interaction group-by-time: p = .016)

Supplementary Figure 2

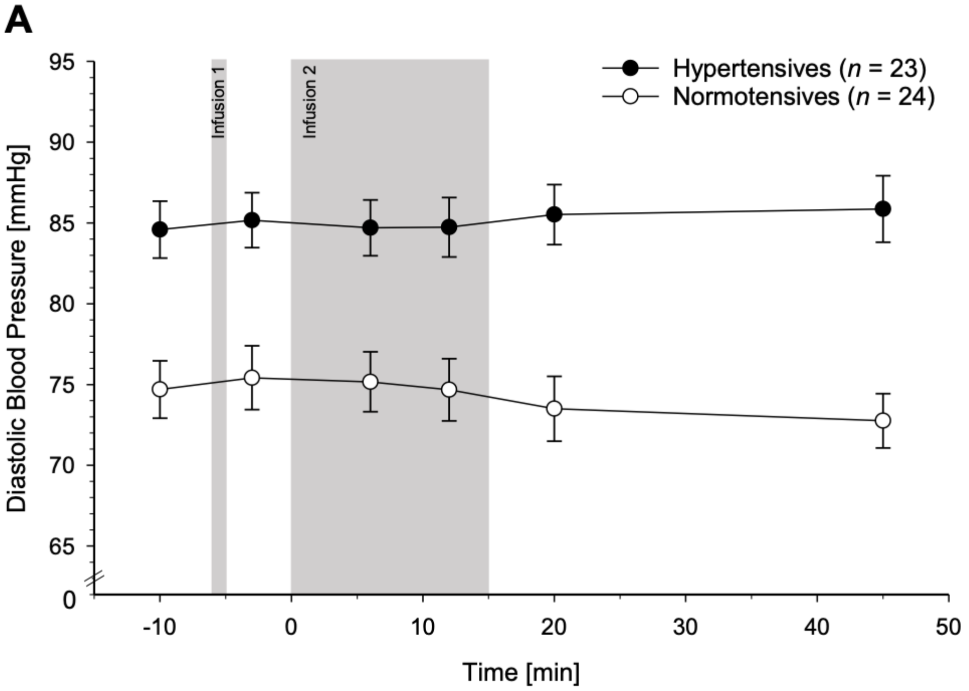

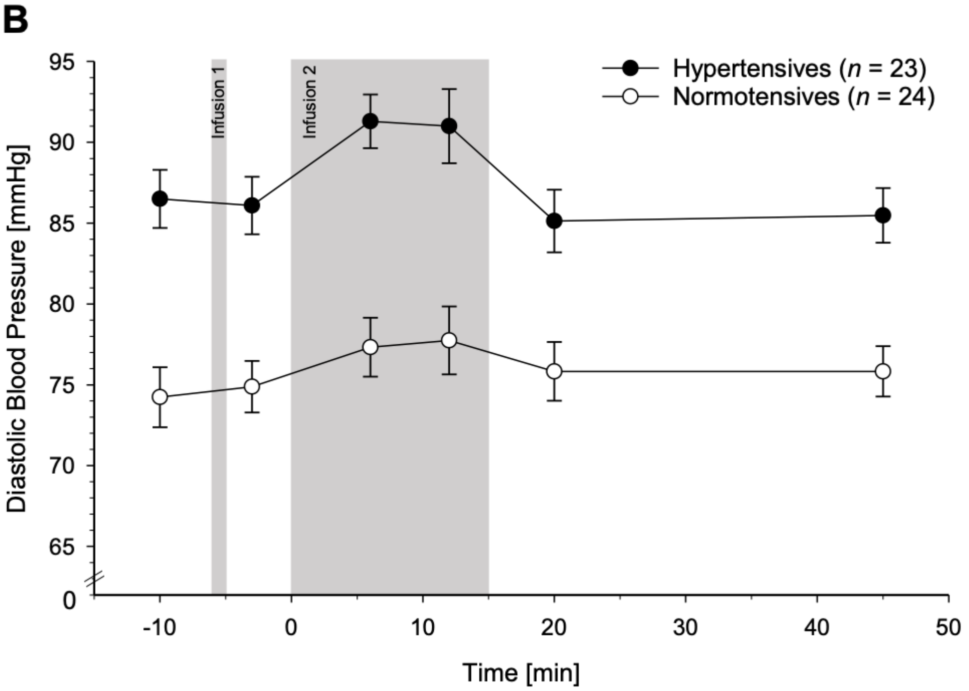

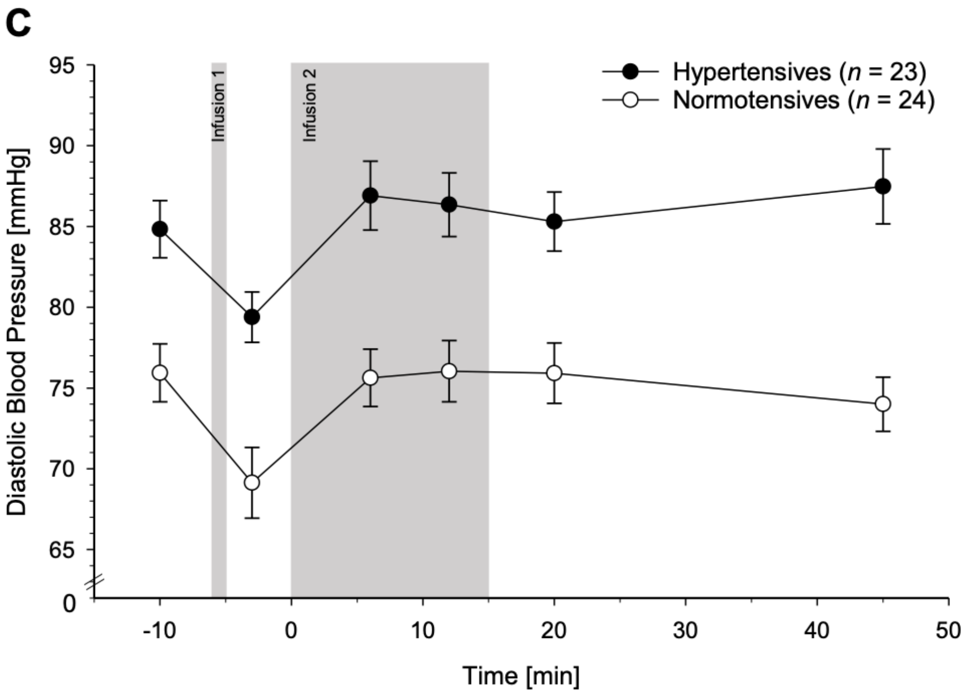

Supplementary Figure 2. Diastolic blood pressure reactivity of hypertensive participants (n = 23) and normotensive controls (n = 24) to the three different substance infusions separately for each trial (mean ± *SEM*). (A) *Trial 1*: saline + saline (repeated measures ANCOVA, interaction group-by-time: p = .29). (B) *Trial 2*: saline + norepinephrine (repeated measures ANCOVA, interaction group-by-time: p = .034). (C) *Trial 3*: non-selective α-adrenergic receptor blocker phentolamine + norepinephrine (repeated measures ANCOVA, interaction group-by-time: p = .26)

Supplementary Figure 3

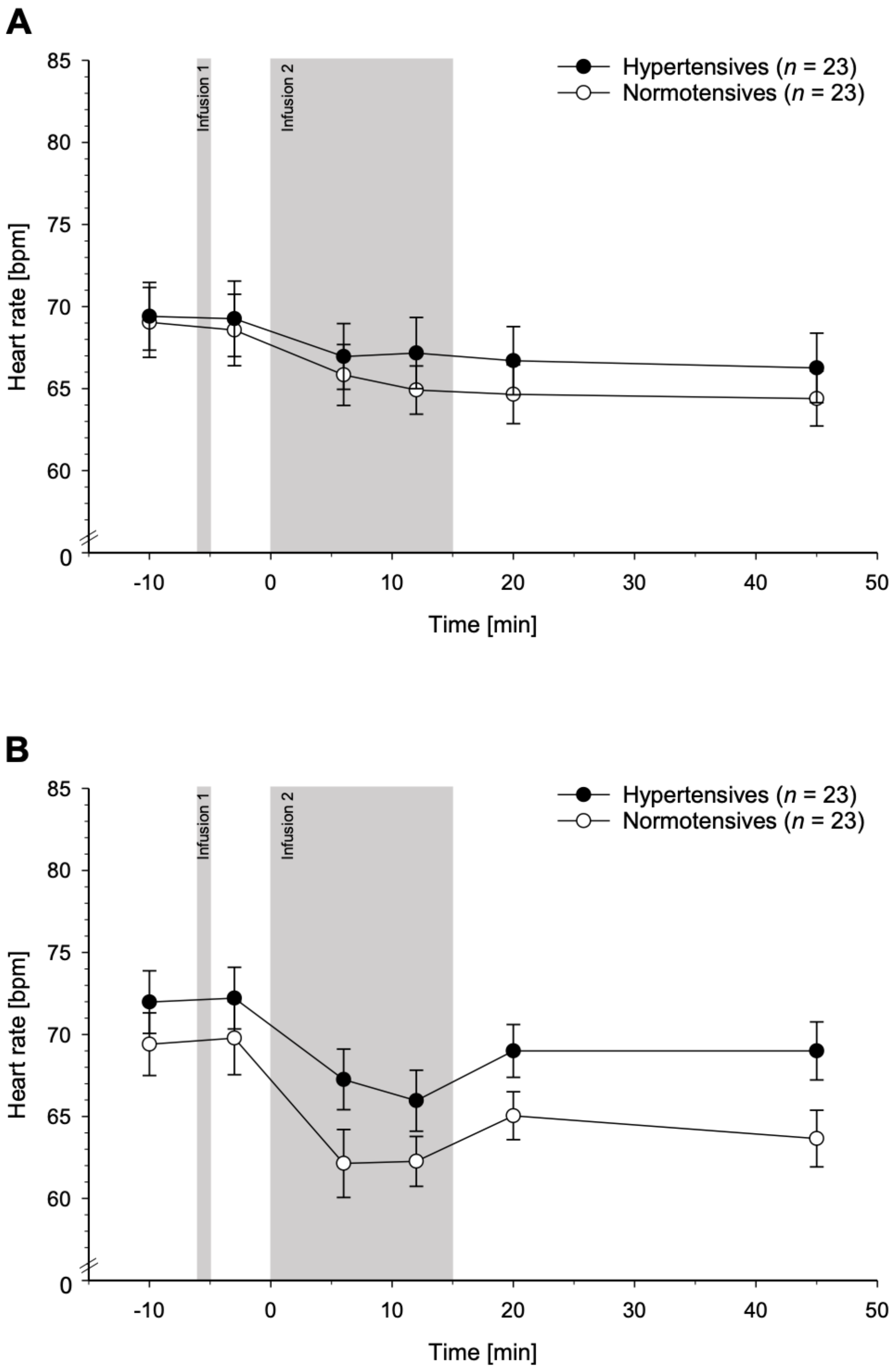

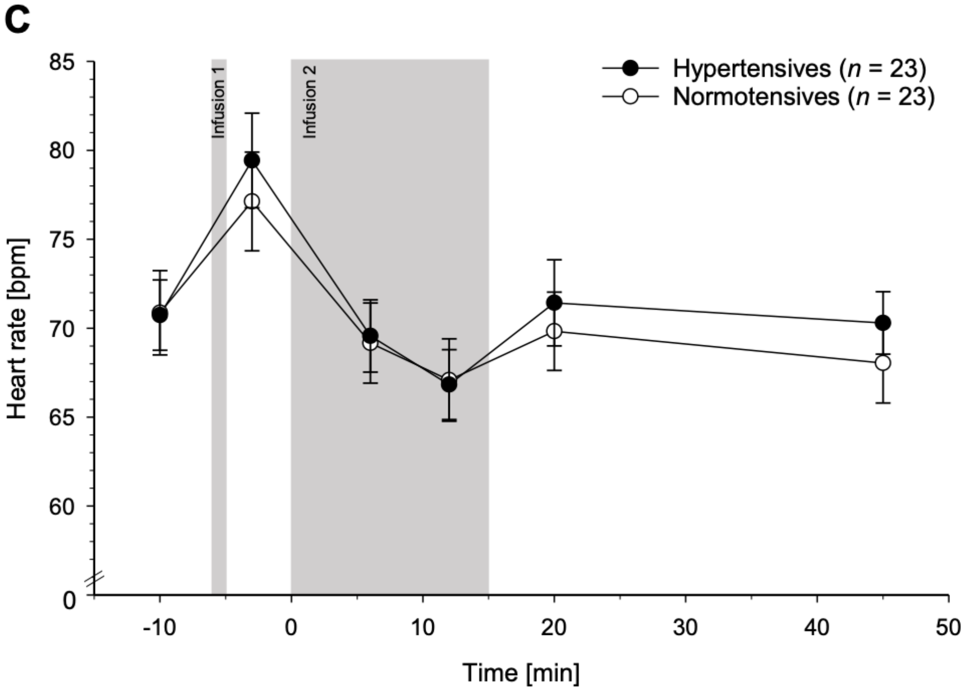

Supplementary Figure 3. Heart rate reactivity of hypertensive participants (n = 23) and normotensive controls (n = 23) to the three different substance infusions separately for each trial (mean ± *SEM*). Heart rate reactivity across the three experimental trials did not differ between HT and NT as revealed by the calculated general linear model (interaction trials-by-group-by-time: p = .73). (A) *Trial 1*: saline + saline. (B) *Trial 2*: saline + norepinephrine. (C) *Trial 3*: non-selective α-adrenergic receptor blocker phentolamine + norepinephrine

2.2 Discussion of Supplementary Figure 1

As depicted in Figure S1B, HT reach the maximum SBP increase in response to saline + norepinephrine at the first measurement timepoint during norepinephrine -infusion and thus earlier as compared to NT controls. The sympathetic overactivity in EHT may set HT on "alert", which may increase preparedness in HT to physiologically react in response to stress or norepinephrine-infusion (5). In reaction to phentolamine + norepinephrine (Figure S1C), the maximum SBP increase in HT is observed at the second measurement timepoint during norepinephrine-infusion comparable with NT who show similar SBP levels during both infusion measurements. The delayed SBP increase in HT after phentolamine + norepinephrine as compared to saline + norepinephrine, might be attributable to the previous BP decline in reaction to the phentolamine-infusion. Also, the attenuation of DBP reactivity to norepinephrine-infusion after phentolamine-infusion in HT may affect SBP reactivity (6).

2.3 Supplementary Tables

Supplementary Table 1. Blood count results for normotensive and hypertensive participants

	Normotensives (n = 24)	Hypertensives (n = 24)	***p***
Creatinine (µmol/L)	79.21 ± 1.55 (68.00 – 93.00)	81.96 ± 1.90 (67.00 – 101.00)	.27
Sodium (mmol/L)	141.38 ± 0.29 (137.00 – 144.00)	141.25 ± 0.39 (139.00 – 148.00)	.80
Potassium (mmol/L)	4.09 ± 0.04 (3.70 – 4.40)	4.04 ± 0.04 (3.70 – 4.50)	.40
HbA1c (%)	5.50 ± 0.08 (4.90 – 6.30)	5.66 ± 0.07, n = 21 (5.00 – 6.40)	.15
TC/HDL-c	3.76 ± 0.21, n = 23 (2.45 – 6.62)	4.02 ± 0.26 (2.22 – 6.84)	.43

Footnote. Values are means ± standard error of the mean (range); HbA1c = hemoglobin A1c; TC = total cholesterol; HDL-C: high-density lipoprotein cholesterol; n = sample size; deviating sample sizes of a parameter are indicated.

3 Supplementary References

1. Larsen R. Kardiovaskuläre Medikamente. Anästhesie und Intensivmedizin für die Fachpflege. 2016:618-26.

2. Martin C, Medam S, Antonini F, Alingrin J, Haddam M, Hammad E, et al. NOREPINEPHRINE: NOT TOO MUCH, TOO LONG. Shock. 2015;44(4):305-9.

3. Ugarte F, Hurtado-Coll A, for the Sildenafil Study G. Comparison of the efficacy and safety of sildenafil citrate (Viagra®) and oral phentolamine for the treatment of erectile dysfunction. International Journal of Impotence Research. 2002;14(2):S48-S53.

4. Padma-Nathan H, Goldstein I, Klimberg I, Coogan C, Auerbach S, Lammers P, et al. Long-term safety and efficacy of oral phentolamine mesylate (Vasomax®) in men with mild to moderate erectile dysfunction. International Journal of Impotence Research. 2002;14(4):266-70.

5. Julius S, Nesbitt S. Sympathetic Overactivity in Hypertension*: A Moving Target. American Journal of Hypertension. 1996;9(S4):113S-20S.

6. Cohn JN. Blood pressure and cardiac performance. The American Journal of Medicine. 1973;55(3):351-61.
